# Mechanistic insights into accelerated α-synuclein aggregation mediated by human microbiome-associated functional amyloids

**DOI:** 10.1016/j.jbc.2022.102088

**Published:** 2022-05-30

**Authors:** Sujeet S. Bhoite, Yilin Han, Brandon T. Ruotolo, Matthew R. Chapman

**Affiliations:** 1 Department of Molecular, Cellular and Developmental Biology, University of Michigan, Ann Arbor, Michigan, USA; 2 Department of Chemistry, University of Michigan, Ann Arbor, Michigan, USA

**Keywords:** Parkinson’s disease, α-synuclein, human microbiome, CsgA, native ion-mobility mass spectrometry, CD, circular dichroism, CCS, collisional cross section, CY CsgA, *Citrobacter youngae* CsgA, CD CsgA, *Cedecea davisae* CsgA, CY^GK^ CsgA, CY Gatekeeper CsgA, EC CsgA, *Escherichia coli* CsgA, GI, gastrointestinal, HA CsgA, *Hafnia alvei* CsgA, HFIP, 1,1,1,3,3,3-hexafluoro-2-propanol, IM-MS, ion mobility mass spectrometry, nESI, nano-electrospray ionization, PD, Parkinson’s disease, TEM, transmission electron microscopy, ThT, Thioflavin-T, YR CsgA, *Yokenella regensburgei* CsgA

## Abstract

The gut microbiome has been shown to have key implications in the pathogenesis of Parkinson’s disease (PD). The *Escherichia coli* functional amyloid CsgA is known to accelerate α-synuclein aggregation *in vitro* and induce PD symptoms in mice. However, the mechanism governing CsgA-mediated acceleration of α-synuclein aggregation is unclear. Here, we show that CsgA can form stable homodimeric species that correlate with faster α-synuclein amyloid aggregation. Furthermore, we identify and characterize new CsgA homologs encoded by bacteria present in the human microbiome. These CsgA homologs display diverse aggregation kinetics, and they differ in their ability to modulate α-synuclein aggregation. Remarkably, we demonstrate that slowing down CsgA aggregation leads to an increased acceleration of α-synuclein aggregation, suggesting that the intrinsic amyloidogenicity of gut bacterial CsgA homologs affects their ability to accelerate α-synuclein aggregation. Finally, we identify a complex between CsgA and α-synuclein that functions as a platform to accelerate α-synuclein aggregation. Taken together, our work reveals complex interplay between bacterial amyloids and α-synuclein that better informs our understanding of PD causation.

Amyloids are highly ordered, fibrous quaternary structures formed by the assembly of protein or peptide monomers into stacked cross β-sheets (1, 2). While there are naturally occurring functional amyloids, amyloids and amyloid formation are more commonly associated with protein misfolding and human diseases (3). Parkinson’s disease (PD) is the second most common neurodegenerative disease in the world with more than 10 million patients (4). The canonical pathophysiological hallmark of PD is the abnormal accumulation of a neuronal protein α-synuclein into insoluble amyloid aggregates which eventually leads to the death of dopaminergic neurons (5). Currently treatments for PD are limited to relieving late-stage symptoms rather than stopping disease progression (6). This has given rise to the need for studying PD pathology at early stages of the disease.

Historically, PD has been mainly studied within the central nervous system. However, it is interesting to note that nonmotor symptoms of PD such as gastrointestinal (GI) dysfunction often precede the onset of motor symptoms by years (7, 8). Nearly 80% of PD patients suffer from constipation and GI dysfunction (9, 10). Braak’s hypothesis states that the abnormal accumulation of α-synuclein amyloid aggregates initiates in the GI tract followed by transmission to the brain *via* the vagus nerve (11, 12). Interestingly, α-synuclein aggregates have been shown to accumulate first in the peripheral sites such as the GI tract before migrating to the brain *via* the vagus nerve (13, 14, 15). It appears that the vagus nerve serves as a bidirectional gateway between the enteric nervous system which innervates the gut and the brain (16). Moreover, emerging new studies have pointed toward truncal vagotomy and removal of the appendix to correlate with a decreased risk of PD in humans (17, 18). All these studies suggest a potential role for the GI tract in PD. This connection is not surprising, given the fact that the gut–brain axis has been shown to be an important factor in many neurological conditions (19, 20). The GI tract is home to a large and diverse ecosystem of microorganisms, which play an important role in various physiological processes (21, 22). Changes in the gut microbiome have been implicated in many disease conditions (23). In the case of PD, several studies have revealed differences in the gut microbiome diversity between healthy individuals and PD patients (24, 25, 26, 27). However, it is still unclear whether this change in the gut microbiome diversity is a cause or effect of PD. In a recent study, Sampson *et al.* (28) reported that in germ-free α-synuclein overexpressing mice, fecal transplant of PD patients promoted α-synuclein–mediated motor deficits and brain pathology compared to the control group. This study for the first time experimentally demonstrated the involvement of the gut microbiome in PD.

The National Institutes of Health Human Microbiome Project identified 823 unique species of bacteria including 72 species from the Enterobacteriaceae family in the GI track (29, 30). Previous studies have shown a higher Enterobacteriaceae abundance in PD patients compared to healthy individuals (24). Bacteria belonging to the Enterobacteriaceae family can produce a functional amyloid called curli (31). In addition to Enterobacteriaceae, the curli operon is widespread among diverse phyla (32). The major component of the curli amyloid fibers is a protein called CsgA (33). CsgA is secreted outside the cell as an unstructured protein *via* a dedicated secretion system (34, 35). Once outside the cell, CsgA aggregates into curli fibers, which help the bacteria in biofilm formation, host cell attachment, and defense against bacteriophages (36, 37, 38). Exposure to *Escherichia coli* curli fibers increases α-synuclein aggregation in the guts and brains of rats and *Caenorhabditis elegans* (39). More recently, a direct link between the gut microbiome and PD was revealed. In germ-free α-synuclein overexpressing mice, colonization with curli producing *E. coli* promoted α-synuclein pathology in the brain and led to enhanced motor symptoms (40). These studies promoted us to ask whether the human gut microbiome harbors curli-producing bacteria from the Enterobacteriaceae family that would influence α-synuclein aggregation.

Here, we report the characterization of CsgA homologs from the human gut microbiome and their role in α-synuclein aggregation. Four gut bacterial CsgA homologs were identified that are encoded by bacteria present in the human microbiome. These four CsgA homologs were cloned, purified, and biochemically assessed for their amyloid-forming properties. The ability of CsgA homologs to accelerate α-synuclein aggregation was correlated to their intrinsic amyloid-forming propensities, wherein slow-aggregating CsgA homologs had a greater ability to accelerate α-synuclein aggregation than fast-aggregating CsgA homologs. Native ion mobility mass spectrometry (IM-MS) was used to probe the oligomeric states of the homologs and average collision cross sections (CCSs) were also measured. We found that the dimeric CsgA species was in a compacted conformation in slow-aggregating CsgA homologs, which correlated with their ability to accelerate α-synuclein aggregation. Based on our data, we propose a mechanism wherein the intermediate metastable species of CsgA homologs can accelerate α-synuclein aggregation. Our study presents a workflow to further understand the interplay between gut bacterial amyloids and PD pathogenesis.

## Results

### Amyloidogenic CsgA-expressing bacteria are present in the human microbiome

**Figure 1:**
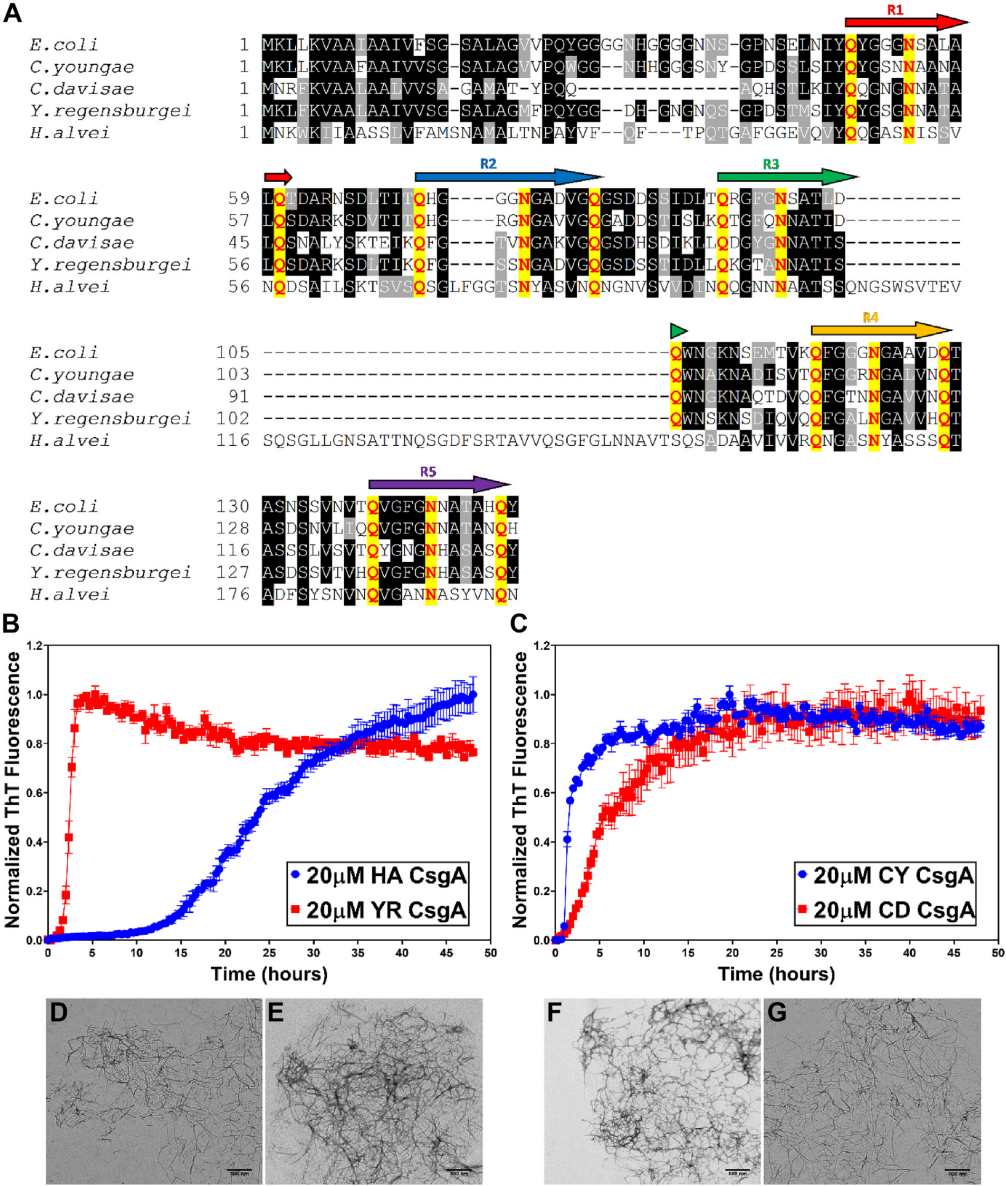
**Gut bacterial CsgA homologs are amyloidogenic in nature.***A*, sequence alignment of CsgA homologs. The five amyloid imperfect repeat units with Q-(X)4-N-(X)5-Q motif are marked as R1 to R5. The Q and N residues in the motifs are marked in *red*. The alignment was done using T-Coffee (http://tcoffee.crg.cat/apps/tcoffee/do:regular) with default parameters and visualized using Boxshade (https://embnet.vital-it.ch/software/BOX_form.html). Aggregation kinetics of CsgA homologs purified from the human microbiome were monitored using Thioflavin T fluorescence over the course of 48 h at 37 °C. *B*, 20 μM HA CsgA (*blue*) and 20 μM YR CsgA (*red*). *C*, 20 μM CY CsgA (*blue*) and 20 μM CD CsgA (*red*). (Error bars represent SEM of three replicates). *D*–*G*, negatively stained transmission electron micrographs of CsgA fibers. Representative images of the samples were taken 48 h postaggregation. *D*, HA CsgA. *E*, YR CsgA. *F*, CY CsgA. *G*, CD CsgA. (The scale bars represent 500 nm). CD CsgA, *Cedecea davisae* CsgA; CY CsgA, *Citrobacter youngae* CsgA; HA CsgA, *Hafnia alvei* CsgA; YR CsgA, *Yokenella regensburgei* CsgA.

*E. coli* CsgA (EC CsgA) is composed of five conserved imperfect repeat units designated R1-R5, each with a Q-X4-N-X5-Q consensus sequence (41). Each repeating unit is predicted to form a strand-loop-strand motif covalently linked to each other to resemble a β-helical structure (42, 43). Mutational studies have revealed the importance of this motif in promoting CsgA amyloid formation (44). To investigate the presence of CsgA homologs in human gut microbiome, we used the reference genomes of bacteria isolated from the GI tract and deposited to the National Institutes of Health Human Microbiome Project (29, 30). This dataset has 823 reference genomes, including 61 unique Enterobacteriaceae family *E. coli* strains. Each unique Enterobacteriaceae species was scanned for CsgA homologs and all the other proteins in the curli operon. For this study, we focused on four CsgA homologs based on their sequence diversity compared to EC CsgA, namely, *Hafnia alvei* (HA CsgA), *Yokenella regensburgei* (YR CsgA), *Citrobacter youngae* (CY CsgA), and *Cedecea davisae* (CD CsgA). HA CsgA displayed the least similarity to EC CsgA with only 31% sequence identity, while CY CsgA shared 67% sequence identity with EC CsgA (Table S1). Despite the sequence diversity within the CsgA homologs, sequence alignment with EC CsgA revealed the presence of the conserved Q-X4-N-X5-Q consensus sequence (Fig. 1*A*).

To characterize the amyloid-forming capabilities of the CsgA homologs from *H. alvei*, *Y. regensburgei*, *C. youngae,* and *C. davisae*, each sequence was cloned into pET28a vector and then expressed and purified as described in the Experimental procedures (Table S3). The ability of each CsgA homolog to adopt an amyloid-like fiber was first assessed using a Thioflavin-T (ThT) fluorescence assay (45, 46). As reported previously (47), EC CsgA fibrillation kinetics show a typical sigmoidal aggregation behavior, wherein the ThT fluorescence increases after a lag time of 2.5 ± 0.5 h at 25 °C (Fig. S1*A*). At 37 °C, the lag time was reduced to approximately 1 h (Fig. S1*A*). Interestingly, compared to EC CsgA, HA CsgA and YR CsgA showed a delayed lag phase of ∼10 h and ∼1.5 h, respectively, while CY CsgA and CD CsgA showed rapid aggregation with a lag phase of ∼0.5 h (Fig. 1, *B* and *C*). The β-sheet–rich amyloid nature of the CsgA homologs was confirmed by far-UV circular dichroism (CD) of the fibers after 48 h of incubation (Fig. S1*B*), and the purity of the cloned CsgA homologs was confirmed by SDS-PAGE gels (Fig. S1, *C–F*) and native IM-MS (see below). We next looked at the fiber morphology with a transmission electron microscopy (TEM) and each of the CsgA homologs assembled into fibers that were similar in appearance to those made by EC CsgA (Fig. 1, *D* and *G*).

### Gut bacterial CsgA homologs assembly into amyloid fibers *in vivo*

**Figure 2:**
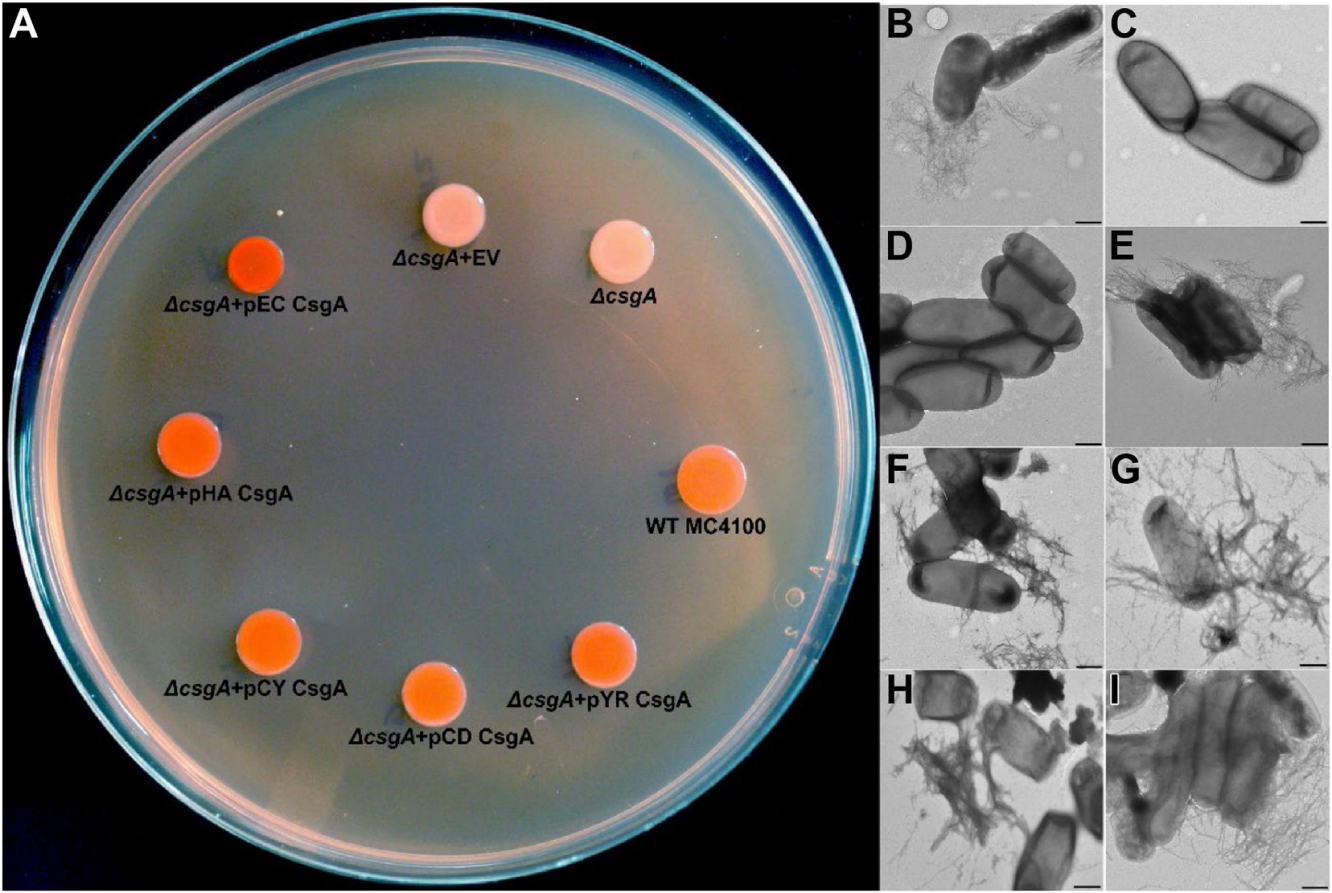
**Gut bacterial CsgA homologs assemble into amyloidogenic fibers *in vivo*.***A*, *Escherichia coli* MC4100 Δ*csgA* cells were transformed with plasmids encoding the different CsgA homologs under the native *E coli csgBAC* promoter and fused to *E. coli* CsgA sec signal and N-terminal 22 amino acids and observed post 48 h incubation on YESCA Congo *red* indicator plates at 25 °C (EV=empty vector). Representative negative-stained transmission electron micrographs of *B*, WT *E. coli* MC4100; *C*, *E. coli* MC4100 Δ*csgA*; *D*, *E. coli* MC4100 Δ*csgA* + EV; *E*, *E. coli* MC4100 Δ*csgA* + pEC CsgA; *F*, *E. coli* MC4100 Δ*csgA* + pHA CsgA; *G*, *E. coli* MC4100 Δ*csgA* + pCY CsgA; *H*, *E. coli* MC4100 Δ*csgA* + pCD CsgA, and *I*, *E. coli* MC4100 Δ*csgA* + pYR CsgA. (The scale bars represent 500 nm). CD CsgA, *Cedecea davisae* CsgA; CY CsgA, *Citrobacter youngae* CsgA; EC CsgA, *Escherichia coli* CsgA; HA CsgA, *Hafnia alvei* CsgA; YR CsgA, *Yokenella regensburgei* CsgA.

To test whether gut bacterial CsgA homologs would form amyloid fibers *in vivo*, *E. coli* cells lacking endogenous CsgA were transformed with plasmids that expressed the CsgA homologs. In *E. coli*, assembly of CsgA fibers is guided by the Type VIII secretion system (34). After CsgA monomers are secreted to the extracellular space, curli amyloid fiber formation is initiated by the outer membrane–associated CsgB nucleator protein (34). CsgA homologs from *Salmonella typhimurium* LT2 and *Citrobacter koseri* have also been shown to be cross-seeded by *E. coli* CsgB both *in vitro* and *in vivo* (48). We thus expressed the CsgA homologs identified from the gut microbiome under the native *E. coli csgBAC* promoter in an *E. coli* MC4100 Δ*csgA* strain (LSR10) (Table S4). To facilitate the extracellular export and assembly, we fused the N-terminal 22 amino acid sequence of EC CsgA (N22) to the different CsgA homologs so that each homolog could be expressed in an *E. coli* Δ*csgA* strain and assessed for its ability to assemble into a curli amyloid fiber on the cell surface. Strains that assemble extracellular amyloid fibers stain red on Congo red indicator plates, while strains that cannot make extracellular amyloid appear in white or light pink (49). WT *E. coli* MC4100 formed red colonies after 48 h incubation at 26 °C (Fig. 2*A*). The Δ*csgA* mutant strain and the Δ*csgA* mutant strain that contained the empty vector pLR2 formed white, nonstained colonies on Congo red indicator plates (Fig. 2*A*). Interestingly, Δ*csgA* strains that harbored plasmids that expressed EC CsgA or any of the gut bacterial CsgA homologs stained red, indicating curli amyloid formation occurred (Fig. 2*A*). Whole-cell TEM revealed the presence of cell membrane–associated curli fibers which were indistinguishable from those produced by WT MC4100 or by MC4100:Δ*csgA* transformed with a pLR5 plasmid that expresses EC CsgA protein (Fig. 2,*B*–*I*). Cell membrane–associated CsgA fibers were also detected in whole cell lysates by Western blot with 1,1,1,3,3,3-hexafluoro-2-propanol (HFIP) treatment (Fig. S2*A*). Interestingly, HA CsgA fibers were detected even without HFIP treatment, indicating the SDS-soluble nature of HA CsgA amyloid fibers. To determine if the Congo red positive fibers produced on the surface of *E. coli* were dependent on the CsgB nucleator protein, we performed a similar complementation assay in an *E. coli* MC4100 Δ*csgBA* (LSR13) background. The absence of red colored colonies (Fig. S2*B*) and cell membrane–associated curli fibers (Fig. S2, *C–J*) when gut bacterial CsgA homologs were expressed in Δ*csgBA* background indicated that CsgA amyloid formation *in vivo* was CsgB dependent and not due to the extracellular aggregation of CsgA homologs.

### Gut bacterial CsgA homologs modulate the amyloidogenic aggregation of α-synuclein

**Figure 3:**
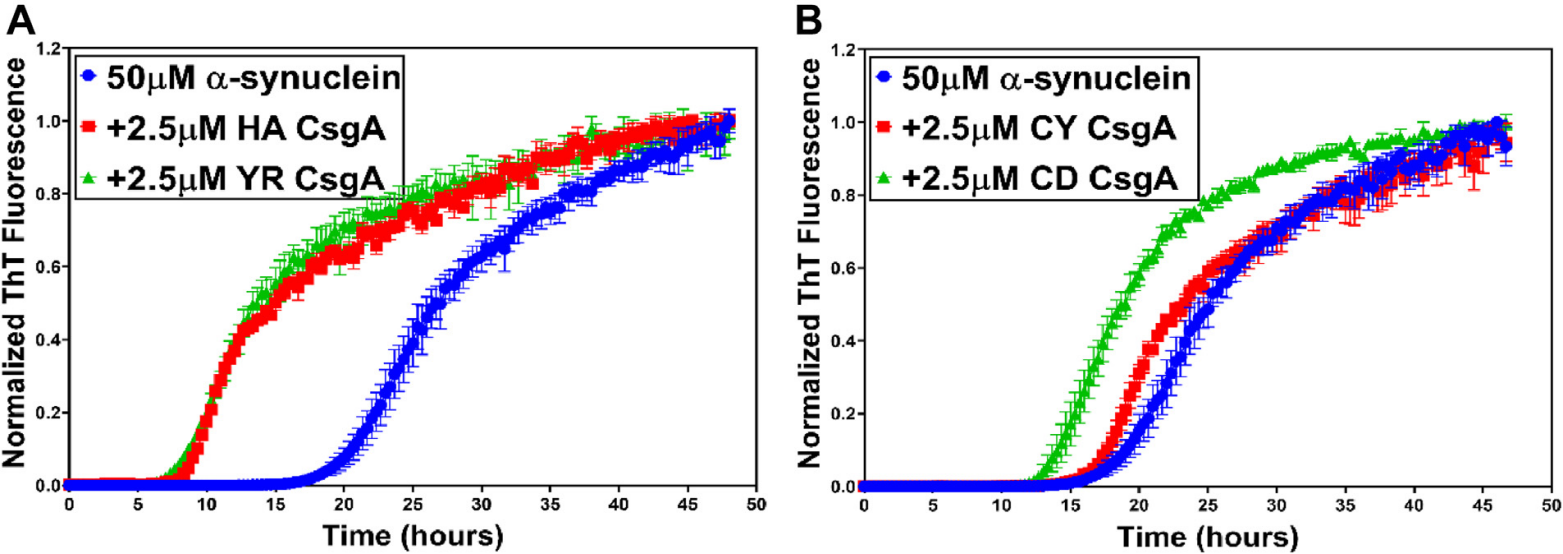
**Gut bacterial CsgA homologs accelerate α-synuclein aggregation.** Aggregation kinetics of α-synuclein alone or in presence of CsgA homologs at 37 °C. *A*, α-synuclein alone (*blue*) or in the presence of HA CsgA (*red*) and YR CsgA (*green*). *B*, α-synuclein alone (*blue*) or in the presence of CY CsgA (*red*) and CD CsgA (*green*). (Error bars represent SEM of three replicates). CD CsgA, *Cedecea davisae* CsgA; CY CsgA, *Citrobacter youngae* CsgA; HA CsgA, *Hafnia alvei* CsgA; YR CsgA, *Yokenella regensburgei* CsgA.

We tested the effect of gut bacterial CsgA homologs on α-synuclein aggregation. EC CsgA has been demonstrated to accelerate α-synuclein amyloid formation (40). The lag phase of α-synuclein aggregation when 50 μM of α-synuclein was mixed with 2.5 μM of EC CsgA (molar ratio of 1.0:0.05 α-synuclein:EC CsgA) was reduced by more than half (Fig. S3*A*). Similarly, in the presence of 2.5 μM HA CsgA and YR CsgA, α-synuclein aggregation was significantly accelerated with the lag phase of aggregation reduced by more than half as compared to α-synuclein alone (Figs. 3*A* and S3, *B* and *D*). Interestingly, at the same concentrations, CY CsgA and CD CsgA did not show significant acceleration of α-synuclein amyloid formation (Figs. 3*B* and S3, *C* and *E*). To investigate this further, we varied the concentration of CsgA homologs and observed the effect on α-synuclein aggregation. We found that the slower aggregating CsgA homologs, HA CsgA and YR CsgA, accelerated α-synuclein aggregation at all the concentrations tested, whereas the faster aggregating CsgA homologs, CY CsgA, and CD CsgA, accelerated α-synuclein aggregation only at low concentration of 0.5 μM, that is, a molar ratio of 1.0:0.01 α-synuclein:CY/CD CsgA. (Fig. S4, *A–H*). The α-synuclein fibers made in the presence of CsgA homologs were analyzed by TEM and far-UV CD. Analysis of the secondary structure by CD revealed minor differences in α-synuclein fibers made in presence or absence of gut bacterial CsgA homologs (Fig. S5*A* and Table S2). In agreement with previous reports (50, 51, 52), TEM under direct magnification of 25,000 to 30,000× showed that α-synuclein fibers formed in the absence of CsgA appeared as rod-like, nontwisted filaments (Fig. S5*B*). The rod-like, nontwisted filaments were also observed in α-synuclein fibers made in the presence of HA CsgA (Fig. S5*C*). Interestingly, α-synuclein fibers generated in the presence of EC CsgA, CY CsgA, and CD CsgA had darkly stained cross bands that suggested that the fibers were twisted (Fig. S5, *D–F*, black arrows).

### Changing the aggregation kinetics of CsgA leads to acceleration of α-synuclein aggregation

**Figure 4:**
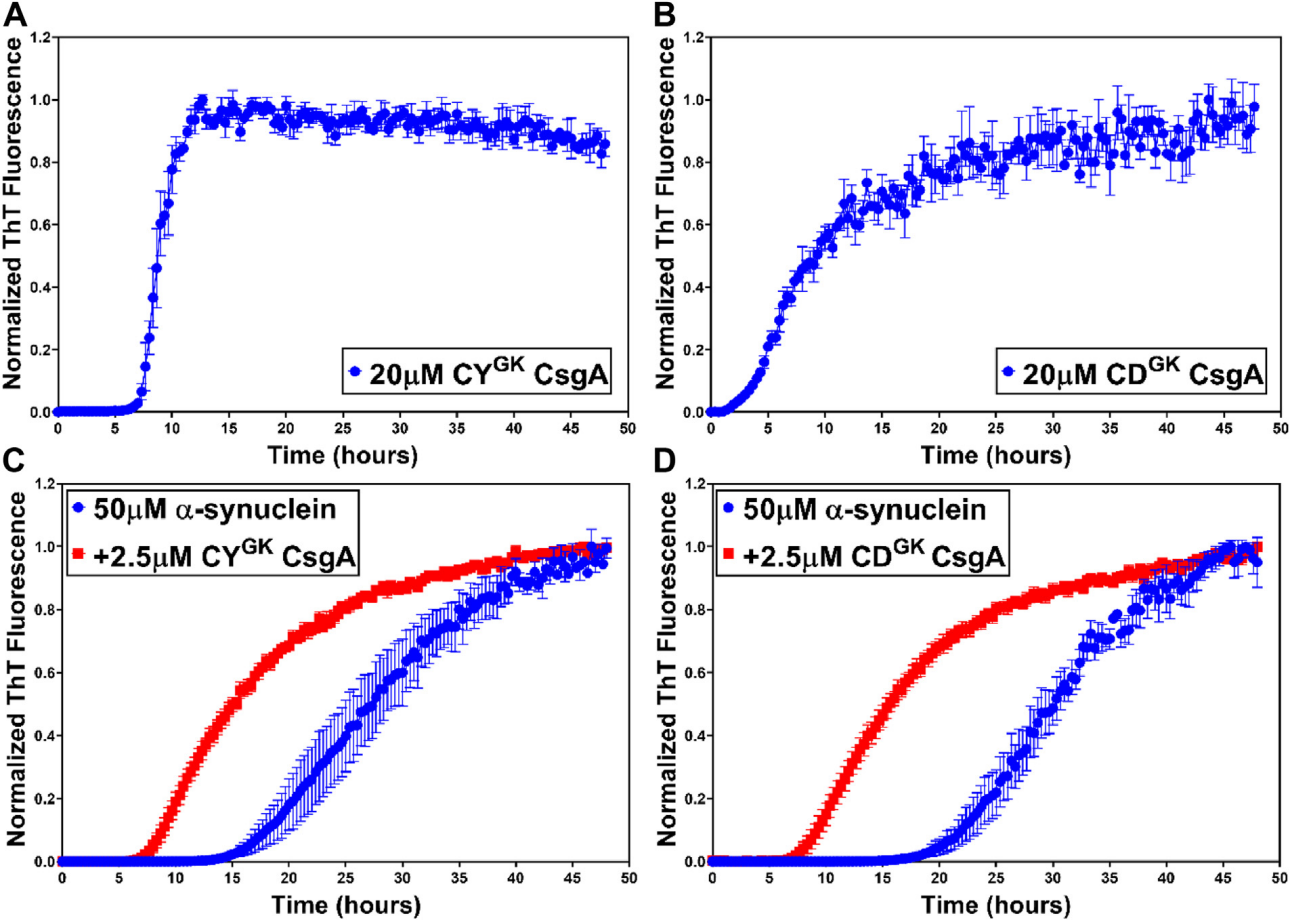
**Gatekeeper CsgA mutants accelerate α-synuclein aggregation.** Aggregation kinetics of Gatekeeper CsgA(CY^GK^ CsgA) variants at 37 °C. *A*, CY^GK^ CsgA. *B*, CD^GK^ CsgA. Aggregation kinetics of α-synuclein alone or in the presence of CY^GK^ CsgA variants. *C*, α-synuclein alone (*blue*) or in presence of CY^GK^ CsgA (*red*). *D*, α-synuclein alone (*blue*) or in presence of CD^GK^ CsgA (*red*). (Error bars represent SEM of three replicates). CY^GK^ CsgA, CY Gatekeeper CsgA.

Our previous work has identified key “gatekeeper” residues in EC CsgA that modulate its assembly into amyloid fibers. Deletion or substitution of these gatekeeper residues in EC CsgA results in faster amyloid aggregation kinetics *in vitro* (53). Sequence alignment of the gut bacterial CsgA homologs showed that HA CsgA and YR CsgA had many of the same gatekeeper residues that EC CsgA has, while CY CsgA and CD CsgA lacked majority of the EC CsgA gatekeeper residues (Fig. S6*A*). The absence of key gatekeeper residues in CY CsgA and CD CsgA might explain the rapid aggregation kinetics of these two CsgA homologs (Fig. 1*B*). Since only slow-aggregating HA CsgA and YR CsgA accelerated α-synuclein aggregation, we hypothesized that the slower aggregating CsgA homologs accelerate α-synuclein aggregation due to low intrinsic amyloid-forming ability compared to the faster aggregating CsgA homologs. To test our hypothesis, we introduced gatekeeper residues into CY CsgA and CD CsgA to slow down their aggregation kinetics. Three amino acid residues in CY CsgA were changed to gatekeeper residues, V78D, S89D, and N125D to get CY Gatekeeper CsgA (CY^GK^ CsgA). In the case of CD CsgA, we substituted the four nongatekeeper residues to gatekeeper residues, K66D, S90D, K77D, and N113D to get CD Gatekeeper CsgA (CD^GK^ CsgA). Strikingly, CY^GK^ CsgA showed delayed amyloid formation as compared to WT CY CsgA with a significantly longer lag phase of ∼5 to 6 h (Fig. 4*A*). In the case of CD^GK^ CsgA, the effect of introducing gatekeeper residues was less significant as the lag phase for amyloid formation changed from ∼0.5 h for WT CD CsgA to ∼2 h for CD^GK^ CsgA (Fig. 4*B*). Morphologically, under the TEM, we observed no difference in the fibril structure between the WT and the gatekeeper variants of CY CsgA and CD CsgA (Fig. S6, *B* and *C*). We hypothesized that the slow-aggregating CsgA homologs would accelerate α-synuclein aggregation. In agreement with our hypothesis, both CY^GK^ CsgA and CD^GK^ CsgA accelerated α-synuclein aggregation at the concentrations tested (Figs. 4, *C* and *D* and S7, *A–F*). Notably, the WT CY CsgA and CD CsgA did not show significant acceleration of α-synuclein aggregation (Fig. 3*B*). Taken together, our results validate our hypothesis that the intrinsic amyloid-forming ability of gut bacterial CsgA dictate their ability to accelerate α-synuclein aggregation.

### IM-MS reveals the presence of a dimeric species in slow-aggregating CsgA homologs

**Figure 5:**
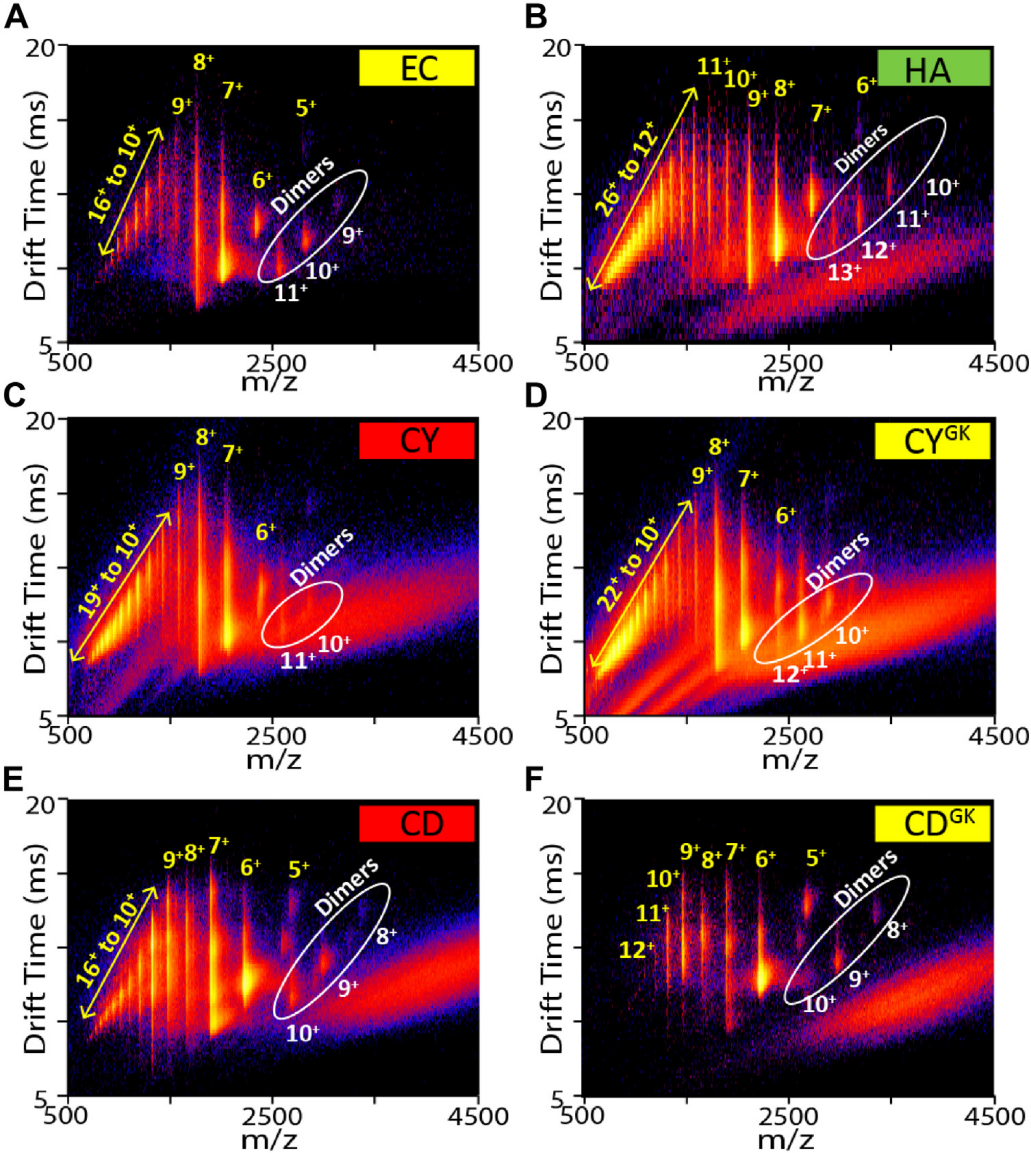
**Multidimensional IM-MS of gut bacterial CsgA homologs reveals the presence of dynamic dimer species.** Charge states were identified for monomeric CsgA (*yellow*) and dimeric CsgA (*white* and *circled*) in both drift time space and in the mass spectra. All homologs were at 20 μM. *A*, EC CsgA. *B*, HA CsgA. *C*, CY CsgA. *D*, CY^GK^ CsgA. *E*, CD CsgA. *F*, CD^GK^ CsgA. CD CsgA, *Cedecea davisae* CsgA; CY CsgA, *Citrobacter youngae* CsgA; CY^GK^ CsgA, CY Gatekeeper CsgA; EC CsgA, *Escherichia coli* CsgA; HA CsgA, *Hafnia alvei* CsgA; IM-MS, ion mobility mass spectrometry.

**Figure 6:**
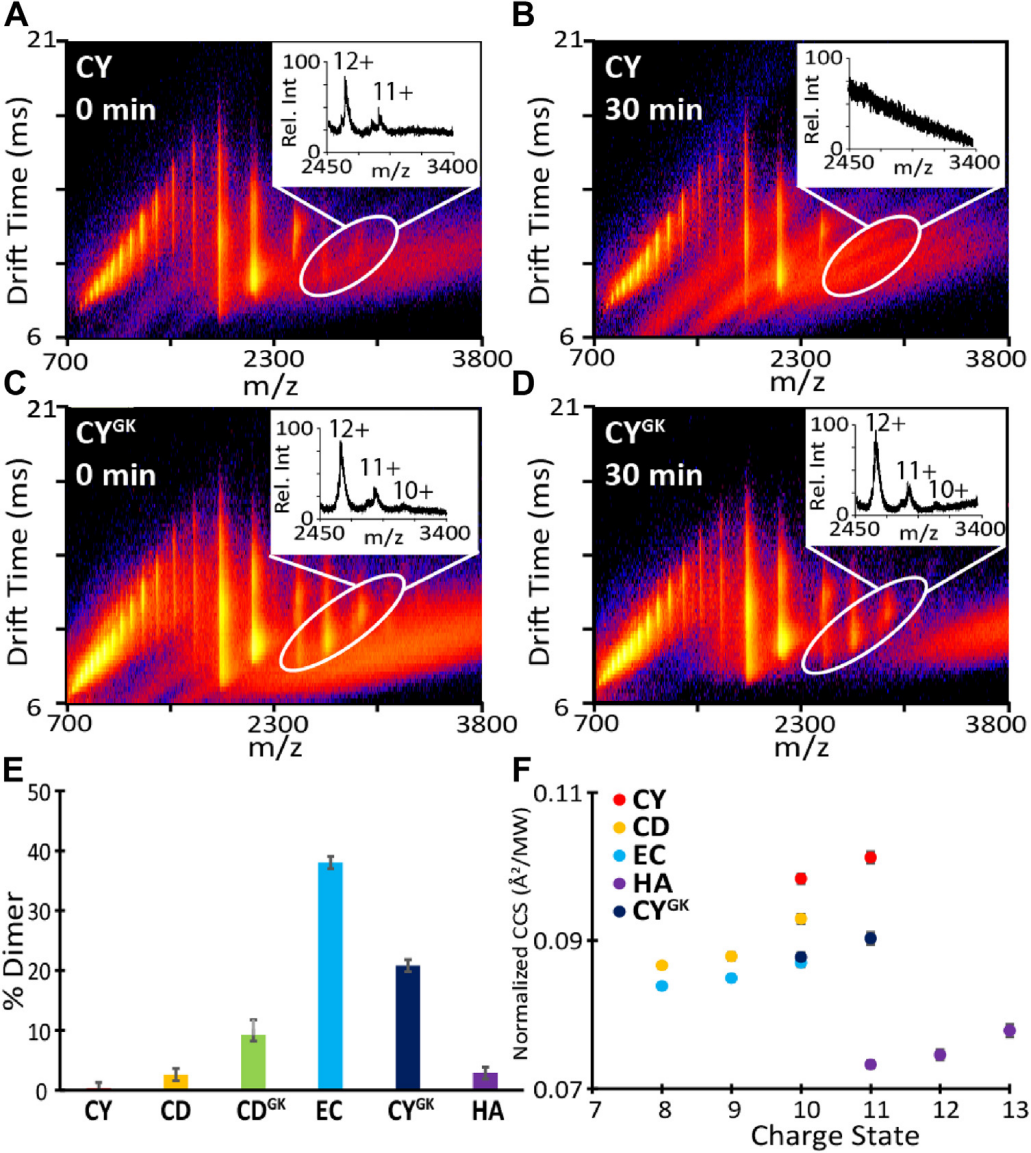
**IM-MS and CCS measurements reveal dimer relations with aggregation kinetics.** Ion mobility data for CY CsgA with enlarged mass spectra inserts featuring 11^+^ and 12^+^ charge states of dimeric CsgA (*white circled*) at *A*, 0 min and *B*, after 30 min incubation. Ion mobility data for CY^GK^ CsgA with enlarged mass spectra inserts featuring 11+ and 12+ charge states of dimeric CsgA (*white circled*) at *C*, 0 min and *D*, after 30 min incubation. *E*, quantification of % dimer of the CsgA variants at 0 min. *F*, normalized CCS values for dimeric CsgA species of all the CsgA variants at 0 min. CCS, collisional cross section; CY CsgA, *Citrobacter youngae* CsgA; CY^GK^ CsgA, CY Gatekeeper CsgA; IM-MS, ion mobility mass spectrometry.

To better understand the mechanism governing the intrinsic amyloid-forming ability of CsgA homologs, the oligomeric species formed during CsgA aggregation were assessed using native IM-MS. Native IM-MS separates proteins in the gas phase and reports on mass to charge (m/z) and rotationally averaged CCS (54). Because of technical difficulties in purifying YR CsgA in large quantities, it was not included in the IM-MS studies. All CsgA homologs namely, EC CsgA, HA CsgA, CY CsgA, and CD CsgA, showed typical mass spectrum for intrinsically disordered protein with a wide range of charge states from 5+ to 26+ (Fig. 5, *A*–*F*) (55, 56, 57). Interestingly, using IM-MS, we observed two distinct populations of monomeric and dimeric CsgA species being present with each of the homolog (Fig. 5, *A*–*F*, white circles). We thus calculated the % dimer at time 0 h for all CsgA homologs (Fig. 6*E*). For EC CsgA, CY CsgA, CD CsgA, CY^GK^ CsgA, and CD^GK^ CsgA, there was a positive correlation between the % dimer detected by IM-MS and the length of the aggregation lag phase (Fig. S8). To investigate this correlation further, we took advantage of the differences in the aggregation kinetics and differences in the ability to accelerate α-synuclein aggregation between WT CY CsgA and CY^GK^ CsgA. We monitored the dimeric species that evolved in these two proteins samples overtime. At 0 h, WT CY CsgA showed five-fold less of dimeric species as compared to CY^GK^ CsgA (Fig. 6, *A* and *C*). After 30 min of incubation, the signal corresponding to dimeric species in CY CsgA had attenuated, while in CY^GK^ CsgA, IM-MS signal associated with the dimeric species persisted (Fig. 6, *B* and *D*). By 45 min of incubation, CY CsgA resulted in sufficient macro-scale aggregates such that our nano-electrospray ionization (nESI) emitters clogged leading to unstable spray that prevented further analysis (Fig. 6*B*). We observed similar trends in the quantities and stabilities of dimeric species when comparing CD CsgA and CD^GK^ CsgA, another homolog pair that differs in aggregation kinetics. At 0 h, WT CD CsgA showed two-fold less of dimeric species as compared to CD^GK^ CsgA (Fig. S9, *A* and *B*). After 30 min of incubation, the dimeric species in CD CsgA significantly decreased in signal intensity, while signal associated with CD^GK^ CsgA dimers remained relatively constant (Fig. S9, *C* and *D*).

We next explored conformational changes in CsgA homologs and the correlation of these conformational changes with their aggregation propensities. Due to variations in the masses of the CsgA homologs, averaged CCS values were normalized against the respective molecular weights of the CsgA homolog to allow for cross comparison (Fig. 6*F*). CCS measurements of dimers formed by CsgA homologs revealed large conformation differences. HA CsgA (purple circles) adopted the most compact CCS among all CsgA homologs, whereas CY CsgA (red circles) exhibited the most extended conformation. In contrast to CY CsgA, CY^GK^ CsgA adopted a more compacted CCS (dark blue circles in Fig. 6*F*), and CY^GK^ CsgA had an increased lag phase in ThT assays (Fig. 4*A*). This is consistent with the previous reports of CCS values of intrinsically disordered proteins, wherein compacted CCS values were correlated with reduced aggregation propensity (58, 59). Taken together, our results suggested that the intrinsic amyloid-forming ability of CsgA was correlated to the amount and conformation of dimers formed during the initial phase of aggregation.

### IM-MS captures weakly bound α-synuclein–EC CsgA complexes in mixtures

**Figure 7:**
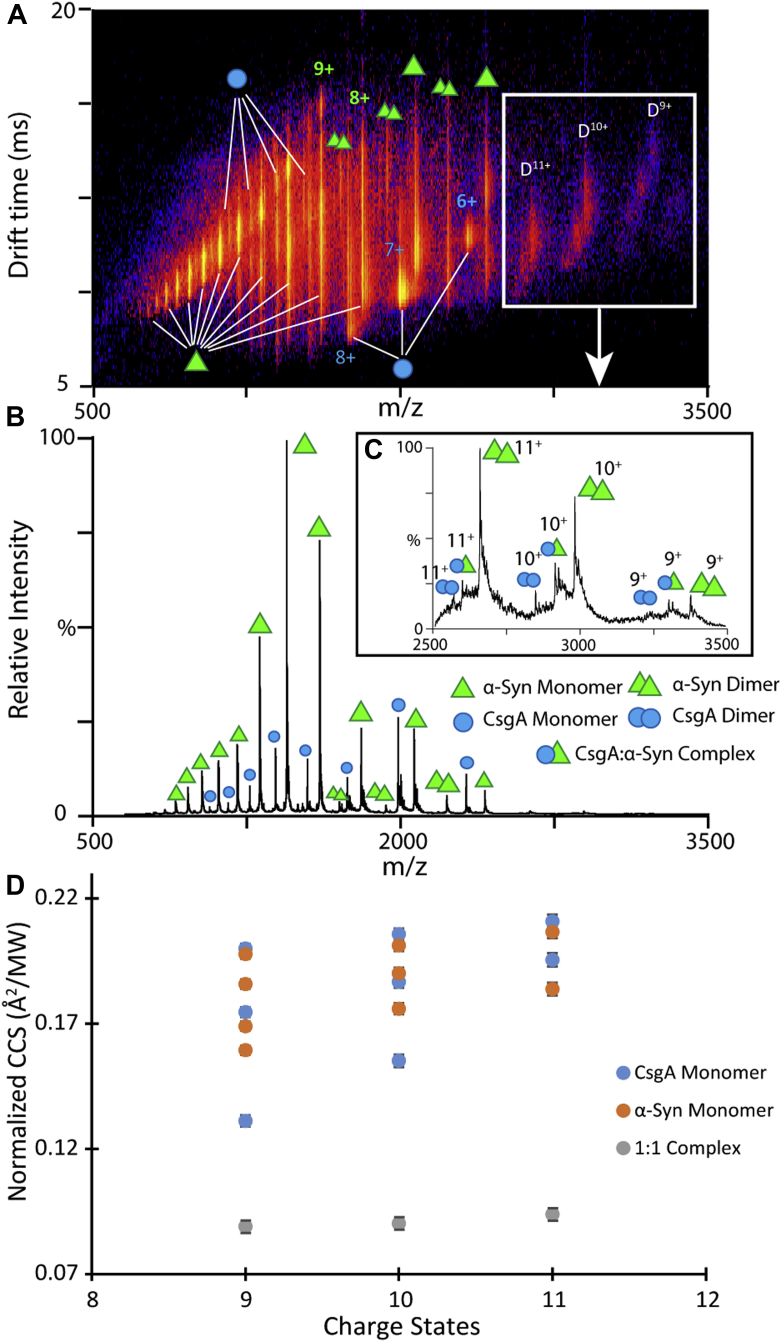
**α-synuclein and CsgA complex formation captured by IM-MS.***A* and *B*, IM-MS data for CsgA incubated with α-synuclein in a 1:1 M ratio for an hour at 37 °C. Monomeric CsgA (*green single triangle*), dimeric CsgA (*green double triangle*), monomeric α-synuclein (*blue single circle*), dimeric α-synuclein (*blue double circle*), and 1:1 α-synuclein:CsgA complexes (*green triangle* and *blue circle*). *C*, magnified MS spectrum of α-synuclein:CsgA complexes flanked on either side by dimeric CsgA and dimeric α-synuclein. *D*, normalized CCS values for monomeric CsgA, monomeric α-synuclein, and α-synuclein:CsgA complex measured by IM-MS. CCS, collisional cross section; IM-MS, ion mobility mass spectrometry.

We next used IM-MS to measure potential interactions between CsgA and α-synuclein. Previous studies have reported noncovalent protein complexes formed with α-synuclein as a binding partner are relatively rare (60, 61, 62, 63). At a molar ratio of 1:1 α-synuclein:EC CsgA, we detected a 1:1 complex of EC CsgA and α-synuclein with charge states ranging from 9^+^ to 11^+^ alongside signals from dimeric CsgA and dimeric α-synuclein (Fig. 7, *A*–*C*). We did not detect interactions between CsgA dimers and α-synuclein but only between CsgA monomers and α-synuclein (Fig. 7*C*). The relative intensity of the complex detected when compared to the signals recorded for unbound α-synuclein and CsgA suggested weak binding interactions between the two proteins. We thus measured the disassociation constant (Kd) for this complex based on a previously reported method from our group that requires the construction of calibration curves that correlate MS signal intensity to protein concentration for both binding partners to account for differences in ionization efficiency (Fig. S10) (64). Following adjustments to MS ion intensity, MS Kd values were extracted from our data and presented in Table 1. The measured Kd value for the 1:1 complex between α-synuclein-CsgA complex was 416 μM ± 96 μM, further suggesting a weak interaction between these two proteins.

**Table 1:** Kd measurement of α-synuclein–CsgA complexes

α-synuclein (μM)	EC CsgA (μM)	Kd (μM)
10	10	489
		298
		320
20	10	429
		551
		412
Avg. Kd. (μM)	416	SD 96

An analysis of normalized CCS values recorded for the protein-binding partners alongside the 1:1 complex revealed significant structure rearrangements of α-synuclein and CsgA upon complex formation. The 9^+^ to 11^+^ charge states of both monomeric EC CsgA and monomeric α-synuclein occupied multiple conformational families as evident by the normalized CCS profiles of these ions (Fig. 7*D*). Interestingly, α-synuclein–EC CsgA complexes adopted a single relatively narrow CCS distribution at equivalent charge states. The 1:1 complex between α-synuclein and CsgA adopted significantly more compact structures than either protein in isolation, producing normalized CCS values approximately half of those recorded for α-synuclein or CsgA alone. Furthermore, we observed that the intensity of the complexes between α-synuclein and CY CsgA at 1:1 M ratio quickly diminished within 30 min of incubation compared to complexes formed between α-synuclein and EC CsgA which remained in the solution for > 30 min (Fig. S11). It is possible that the relatively fast-aggregating CY CsgA self-associates (Fig. 6, *A* and *B*), thus reducing the CY CsgA monomer population available to interact with α-synuclein.

## Discussion

PD is a complex disease that includes a potential role in the gut microbiome in initiating idiopathic PD cases (65). Analysis of the human gut microbiome sequences deposited at the National Institutes of Health Human Microbiome Project revealed the presence of CsgA-encoding bacteria. In the 823 reference genomes deposited in this dataset, there are 72 unique strains belonging to the Enterobacteriaceae family. In PD patients’ gut flora, the level of bacteria belonging to Enterobacteriaceae family has been shown to be elevated compared to healthy individuals (24). Thus, we focused on four CsgA homologs encoded by bacteria belonging to the Enterobacteriaceae family. *In vitro* analysis revealed the amyloidogenic nature of the CsgA homologs. Interestingly, the homologs displayed diverse aggregation kinetics (Fig. 1, *B* and *C*). TEM analysis revealed that the CsgA homologs formed fibers that were indistinguishable from those formed by EC CsgA (Fig. 1, *D–G*). Owing to the diversity in aggregation kinetics, the CsgA homologs we studied could serve as tools to study differences in protein folding and amyloidogenesis among closely related proteins.

Despite the differences in sequences and aggregation kinetics, all gut bacterial CsgA homologs formed cell surface–associated fibers in an *E. coli* Δ*csgA* strain as seen by the red coloration on Congo red plates, TEM analysis, and whole cell western blots (Fig. 2, *A*–*I* and S2*A*). Whole cell western blots also revealed the SDS-soluble nature of HA CsgA fibers. The mature fibers of all other CsgA homologs except HA CsgA required HFIP treatment to depolymerize and migrate on SDS PAGE gels. In the case of HA CsgA, the third imperfect repeat unit containing the Q-X4-N-X5-Q consensus sequence is interrupted by other amino acid residues (Fig. 1*A*). This interruption in one of the repeat units might explain the SDS-soluble nature of HA CsgA fibers as mature fibers of EC CsgA lacking the R3 repeat unit have been observed to be SDS soluble (66). Gut bacterial CsgA homologs assembled into cell surface–associated amyloid fibers on *E. coli* cells. This is in agreement with a previous study showing that CsgA homologs from other species can be cross-seeded by *E. coli* CsgB nucleator protein (48). In nature, most bacteria often reside in multispecies biofilms (67, 68). The human gut is an ideal ecosystem for bacterial biofilm formation, and gut bacterial biofilms have been shown to have an impact on human health (69, 70). Since gut bacteria share the same ecological niche, it is plausible that CsgB proteins from gut bacteria could also cross-seed diverse CsgA homologs and build a heterogenous matrix to form a multispecies biofilm.

The similarities and the dissimilarities in the biophysical characteristics of gut bacterial CsgA homologs prompted us to investigate their effect on α-synuclein aggregation. We observed accelerated α-synuclein aggregation in presence of HA CsgA and YR CsgA at all the concentration we tested, that is, 0.5 μM, 2.5 μM, and 5 μM (Figs. 3*A*, S3*B* and S4, *A* and *C*). Our observations were in agreement with previously published report, detailing the effect of EC CsgA on α-synuclein aggregation (40). In contrast, we detected accelerated α-synuclein aggregation only in the presence of low concentrations (0.5 μM) of CY CsgA and CD CsgA (Fig. S4, *E* and *G*). To understand the relationship between intrinsic aggregation propensities of CsgA homologs and their ability to accelerate α-synuclein aggregation, we modulated the aggregation kinetics of CY CsgA and CD CsgA by introducing gatekeeper residues. In agreement with a previously published report that focused on EC CsgA (53), the substitution of nongatekeeper residues with gatekeeper residues resulted in the retardation of CY CsgA and CD CsgA aggregation (Fig. 4, *A* and *B*). We named these mutants as CY^GK^ CsgA (CY CsgA^V78D/S89D/N125D^) and CD^GK^ CsgA (CD CsgA^K66D/S90D/K77D/N113D^) (Fig. S6*A*). The effect of gatekeeper residues on the aggregation kinetics of CY CsgA was significantly large compared to that of CD CsgA, suggesting the involvement of other amino acid residues in controlling the aggregation kinetics of CD CsgA. Unlike CY CsgA and CD CsgA, CY^GK^ CsgA and CD^GK^ CsgA both accelerated α-synuclein aggregation at all the concentrations tested, that is, 0.5 μM, 2.5 μM, and 5 μM (Figs. 4, *C* and *D* and S7, *A, B, D* and *E*). To summarize, we observed that slow-aggregating CsgA homologs (HA CsgA and YR CsgA) and slow-aggregating mutants (CY^GK^ CsgA and CD^GK^ CsgA) were able to accelerate α-synuclein aggregation at all concentration tested, while fast-aggregating homologs (CY CsgA and CD CsgA) accelerated α-synuclein aggregation only at low concentrations. We therefore hypothesize that at higher concentrations, monomers/oligomers of fast-aggregating CsgA homologs are rapidly integrated into the growing fiber making them less available to interact with free α-synuclein compared to monomers/oligomers of slow-aggregating CsgA homologs. Our hypothesis is supported by a recent study wherein reducing the amyloidogenicity of bacterial functional amyloid FapC increased its ability to inhibit α-synuclein aggregation (71). It was suggested that lowering the intrinsic amyloid-formation propensity of FapC makes the intermediate oligomers more available to interact with α-synuclein, whereas the WT FapC owing to its rapid aggregation immediately forms mature fibers before it even encounters free α-synuclein.

To test the hypothesis that the monomers or small oligomers of fast-aggregating CsgA homologs are too rapidly integrated into the growing fiber, which makes them less available to interact with free α-synuclein, we turned to native IM-MS which allowed us to probe the monomeric and oligomeric species of the CsgA homologs. All CsgA homologs tested displayed large variation in % dimer (Figs. 5, *A*–*F* and 6*E*). The IM-MS data revealed that prior to fibril formation, the CsgA structures and oligomeric states are significantly different that likely results in the altered fibril formation kinetics observed. The rapid decay of CY dimers after 30 min of incubation combined with the low % dimer and short lag time of aggregation indicated that we were indeed capturing dimer species that are on pathway to become higher order oligomers, which eventually become insoluble fibrils (Fig. 6, *A*, *B* and *E*). This difference in % dimer was especially pronounced when comparing WT CsgA homologs with their gatekeeper mutants (*e.g.*, CY and CY^GK^, CD and CD^GK^), as the aggregation propensity is reduced in these mutants. We observed an increase in the relative amounts of the dimer detected for CsgA gatekeeper mutants compared to their respective WTs (Figs. 6, *C*–*E* and S8). The exception to this trend was HA CsgA that exhibits a long lag time and a relatively small % dimer, possibly because dimer formation for HA CsgA is delayed and not picked up during the initial IM-MS experiments (Fig. 6*E*). A more thorough time course experiment revealed a stable dimeric HA CsgA population that increased in distribution throughout the first few hours of incubation prior to doing the IM-MS analysis (Fig. S12). This led us to hypothesize that the dimers formed by HA CsgA are of a different conformation compared to dimers formed by faster aggregating homologs such as CY CsgA. To assess if there were any detectable structural changes between the CsgA homologs during the early steps in aggregation, the IM-MS data were used to generate CCS values that give structural constraints related to dimer size and shape (72, 73). We determined the dimer CCS values across all dimer charge states observed for the six homologs and then normalized these measurements against the respective molecular weights of the homologs (Fig. 6*F*). In comparing the CCS values of WT CsgA homologs, we noted that HA CsgA dimers were substantially more compact than those formed by CY CsgA, CD CsgA, and EC CsgA, thereby supporting our hypothesis that dimers of HA CsgA adopted different conformations when compared with other homologs (Fig. 6*F*). We then compared the normalized CCS values among the CsgA homologs and noted a positive correlation between normalized CCS and aggregation kinetics, indicating that larger normalized CCS values correlated with fast-aggregation kinetics (compare Figs. 6*E* and 1, *A* and *B*). Past reports have linked large CCS values recorded by IM-MS to increased aggregation propensity in a wide range of amyloidogenic systems, and these prior reports align well with our observations (59, 74, 75). The CCS values for the 10^+^ dimer of CY CsgA indicated a larger and more extended conformation than that was measured for CD CsgA. CY CsgA also had a shorter lag time of aggregation than CD CsgA. Lastly, the CCS of CY^GK^ CsgA was smaller than that of WT CY CsgA, despite both the proteins having very similar molecular weights. CY^GK^ CsgA had a longer lag time of aggregation compared to CY CsgA. This further validated the hypothesis that the larger CCS values indicate a more extended dimeric conformation that leads to a more aggressive aggregation propensity.

To investigate the role of dimeric CsgA species in accelerating α-synuclein aggregation, we coincubated α-synuclein with slow- and fast-aggregating CsgA homologs. In slow-aggregating CsgA homologs such as EC CsgA, we observed a 1:1 complex between α-synuclein and CsgA rather than 1:2 complex, suggesting that the dimeric species of CsgA were not directly responsible for accelerating α-synuclein aggregation (Fig. 7*C*). Native IM-MS captured the 1:1 complex between α-synuclein and CsgA (Fig. 7*C*) and not a complex between α-synuclein and dimeric CsgA. In slow-aggregating CsgA homologs, the dimer concentration was higher (potentially leaving fewer free monomers), but the significantly longer lag time of aggregation would suggest that free monomers would still be available to interact with α-synuclein. In contrast, for the fast-aggregating CsgA homologs, the free monomers would be rapidly incorporated into mature fibers making them unavailable for interaction with α-synuclein. Furthermore, very low concentrations of CY CsgA and CD CsgA were able to accelerate α-synuclein aggregation (Fig.S4, *E* and *G*). The CY and CD CsgA homologs would be less likely to self-aggregate at lower concentrations, which might allow the CY and CD monomers to persist for a longer time (76, 77, 78, 79). Thus, at higher concentrations, the fast-aggregating CsgA homologs rapidly form stable fibers by incorporating free monomers into fibrils, while at lower concentrations, the monomers might be available to interact with α-synuclein leading to accelerated aggregation of α-synuclein.

### Proposed mechanism for cross interactions between α-synuclein and CsgA

**Figure 8:**
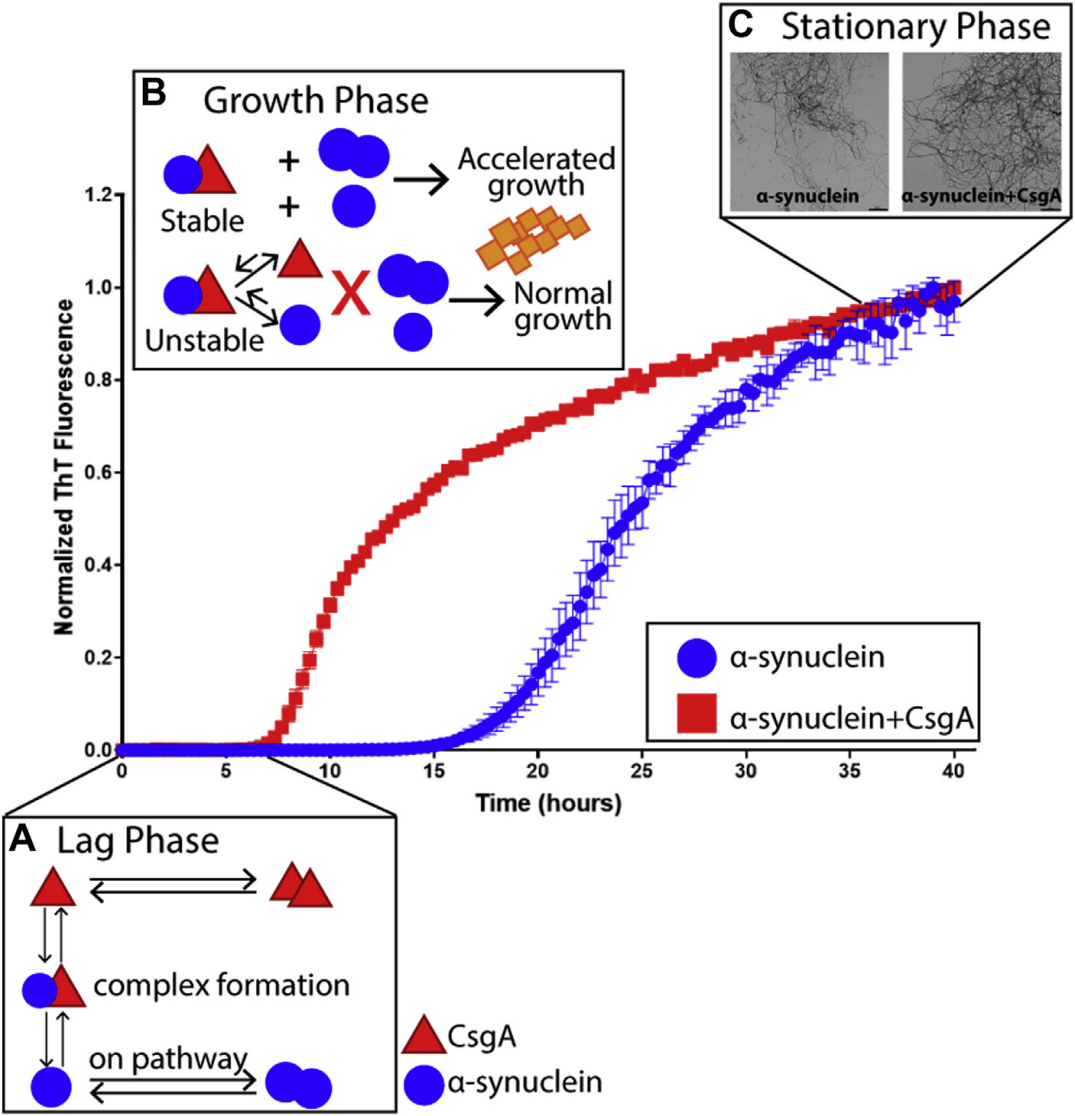
**Proposed mechanism of the interaction between α-synuclein and CsgA.***A*, during the lag phase, monomers of CsgA (*red triangles*) and α-synuclein (*blue circles*) could potentially interact with each other, forming a 1:1 complex. *B*, during the growth phase, the complex can act as a platform for further aggregation or dissociate back to monomer forms of the two proteins. *C*, in the stationary phase, the mature α-synuclein fibrils formed in the presence of CsgA can have a slightly different morphology from those formed in the absence of CsgA (The scale bars represent 500 nm).

Based on our data, a general mechanism for the CsgA and α-synuclein interaction is presented in Figure 8. The interaction that leads to accelerated α-synuclein fibril formation starts in the lag phase where monomers of α-synuclein (red triangles) and monomers of CsgA (blue circles) proceed to form dimers and higher oligomers (Fig. 8*A*). At the same time, a small number of the two proteins also form mixed assemblies that are weakly bound and conformationally compact and act as seeds or nuclei for further fibril formation (Fig. 7, *C, D* and *E* and 8). During the aggregation growth phase, if the nuclei that arise through CsgA and α-synuclein complex formation are comparatively stable (like EC CsgA and α-synuclein compared to CY CsgA and α-synuclein), they can provide a platform for more α-synuclein monomers, dimers, and higher oligomers to assemble on, leading to accelerated fibril growth (Fig. 8*B*). However, if such nuclei are unstable (such as the case with CY CsgA and α-synuclein) (Fig. S11), they can readily dissociate to form monomeric CsgA and monomeric α-synuclein and thus lack the ability to accelerate α-synuclein fibril growth. Mature α-synuclein fibrils formed in the presence of EC, CD and CY CsgA are morphologically distinct from the α-synuclein fibrils formed in the absence of CsgA, or in the presence of HA CsgA (Fig. 8*C* and Fig. S5, *B–F*). Each of the CsgA homologs tested can be placed in either a “slow” or ”fast” aggregating category. The aggregation propensities of the CsgA homologs correlate with the number of dimeric species formed during aggregation. The slow-aggregating CsgA homologs formed more dimers than fast-aggregating CsgA homologs. The slow-aggregating CsgA homologs also have more propensity to accelerate α-synuclein amyloid formation than fast-aggregating CsgA homologs by forming a weakly bound 1:1 complex with α-synuclein and providing a stable nucleus for the accelerated growth of α-synuclein fibrils

In this report, we have provided evidence that CsgA homologs from the human gut microbiome form functional amyloid fibers *in vitro* and *in vivo*. We demonstrate the mechanism which governs the varied aggregation kinetics displayed by the CsgA homologs. These CsgA homologs also interact with α-synuclein leading to accelerated aggregation of α-synuclein. Our data demonstrates that a 1:1 complex between CsgA and α-synuclein can act as a seed to catalyze the rapid aggregation of α-synuclein. Previously, we reported that the colonization of EC CsgA accelerated α-synuclein pathology in mice overexpressing α-synuclein and can cause motor impairment (40). Here, the conformationally compact seed complex observed between EC CsgA and α-synuclein provides an explanation into the interaction mechanism between the two proteins that was previously unknown. EC CsgA is not the only example of bacterially produced amyloid, and as we have indicated in this study, there is a distinct possibility that other CsgA homologs produced by the human gut microbiome can influence α-synuclein aggregation *in vivo*. Our data on these fast and slow categories of homologs and their mutant variants showcases how CsgA aggregation can be modulated in nature. This not only lays the foundation for future *in vivo* studies investigating the role of the gut microbiome in neurodegenerative diseases but also provides potential new drug targets.

## Experimental procedures

### Protein purification

Recombinant human α-synuclein was expressed in *E. coli* and purified as described previously (40). CsgA from *E. coli* was purified as described earlier (49). Gut bacterial CsgA homologs were cloned into pET28a vector for expression using the primers as mentioned in Table S3 and purified as described earlier (49) with certain modifications. Briefly, cell pellets of CY CsgA, CY^GK^ CsgA, CD CsgA, and CD^GK^ CsgA were first treated with 2 ml HFIP and incubated at room temperature for 10 min followed by routine CsgA purification as described in (49).

### Transmission electron microscopy

For CsgA TEM, 20 μM of protein was incubated at 37 °C for 48 h. Ten microliter aliquots were taken at 0 h or 48 h and spotted on formvar-coated copper grids, incubated for 5 min, and washed with MilliQ water before staining with 1% uranyl acetate solution for 5 min. For α-synuclein fibers made in the presence of CsgA, after 48 h of incubation at 37 °C, the reaction was diluted to 10 μM using 50 mM potassium phosphate buffer; pH 7.4. Ten microliter of the sample was spotted on formvar-coated copper grids, incubated for 5 min, and washed with MilliQ water before staining with 1% uranyl acetate solution for 5 min. For whole-cell imaging, *E. coli* MC4100 cells expressing CsgA homologs were scrapped from the YESCA-agar plates and resuspended to 1.0 A_600nm_ in 50 mM potassium phosphate buffer pH 7.4 before applying 10 μl of the cell suspension to formvar-coated grids followed by staining with 1% uranyl acetate solution. Samples were imaged on the Jeol electron microscope (JEOL1400plus, JEOL Ltd).

### Circular dichroism

Protein samples of 20 μM for CsgA and 50 μM for α-synuclein in 50 mM potassium phosphate buffer pH 7.4 were analyzed using a Jasco Corporation made J-810 spectropolarimeter from 190 nm to 260 nm at room temperature immediately after purification and after 48 h incubation at 37 °C.

### Complementation assay

*E. coli* MC4100 *ΔcsgA* cells were transformed with empty vector or plasmid expressing the different CsgA homologs under the native curli *csgBAC* promoter. Overnight grown cultures were diluted to 1.0 A_600nm_ and 4 μl were spotted on YESCA agar (yeast extract, casamino acids) plates supplemented with 50 μg/ml Congo red and incubated at 26 °C for 48 h to induce CsgA expression. Images were recorded using a Canon EOS Rebel XSi camera and the background Congo red color was edited out in Adobe Photoshop (https://www.adobe.com/in/products/photoshop.html).

### ThT assay

The aggregation kinetics of CsgA homologs and the effect of CsgA homologs on α-synuclein aggregation were monitored in black flat-bottom 96-well plates using fluorescent dye ThT in an automated microtiter plate reader (Tecan Infinite M200, TECAN). Freshly purified CsgA homologs were diluted to a final concentration of 20 μM in 50 mM potassium phosphate buffer, pH 7.4. The samples were incubated at 37 °C under quiescent conditions in the presence of 20 μM ThT. The ThT fluorescence intensity was recorded after every 20 min with orbital shaking for 5 s before the readings (excitation: 438 nm; emission: 495 nm). For α-synuclein aggregation kinetics, 50 μM of protein in 50 mM potassium phosphate buffer pH 7.4 with 100 mM NaCl and 20 μM ThT was incubated at 37 °C with constant orbital shaking, and ThT fluorescence intensity was recorded after every 20 min (excitation: 438 nm; emission: 495 nm). To facilitate α-synuclein aggregation, a 2 mm glass bead was added to each well. To study the effect of CsgA homologs on α-synuclein aggregation, freshly purified CsgA homologs were added to each α-synuclein containing wells at different concentrations and the aggregation kinetics was monitored for 48 h. The buffer or CsgA-alone values were subtracted from sample values and are reported as normalized ThT fluorescence. All experiments were performed in triplicates with at least three biological replicates, and the lag phase was calculated by using the following equation (76).

$$y=y_{0\;}+\frac{a}{\left[1+EXP\left(-\frac{x-x_{0}}{k}\right)\right]}$$

Where y_0_ is the initial value of y at time zero, a is the final value of y at the end of the reaction, x_o_ is the value of y at midpoint, and k is the apparent growth rate.

### Gel electrophoresis and Western blot

Overnight grown cultures were diluted to 1.0 A_600nm_ and 4 μl were spotted on YESCA agar (yeast extract, casamino acids) plates. After 48 h incubation at 26 °C, the cells were harvested and resuspended in 50 mM potassium phosphate buffer pH 7.4 and diluted to 0.1 A_600nm_. The harvested cells were duplicated by centrifugation. One duplicate was resuspended in 4× SDS-loading buffer, while the other was treated with HFIP for 10 min at room temperature, dried in SpeedVac (Thermo Fisher Scientific) and then resuspended in 4× SDS-loading buffer. All the samples were run on 15% SDS PAGE gels and transferred to PVDF membrane for Western blot. Blots were probed with anti–CsgA antibody (1:150,000). The blots were imaged on the LI-COR Biosceinces made Odyssey Fc Imager.

### Sample preparation for IM-MS

Purified CsgA from *E. coli* and other CsgA homologs from the gut microbiome were buffer exchanged into 20 mM ammonium acetate (pH 7.4) using Thermo Fisher Scientific Zeba Spin Desalting Columns 7k MWCO. The protein concentration after buffer exchange was assayed using a Thermo Fisher Scientific Pierce Rapid Gold BCA Protein Assay Kit. Samples were diluted to 20 μM with 20 mM ammonium acetate (pH 7.4) for IM-MS experiments and incubated at 37 °C. For the study of protein–protein binding, purified α-synuclein was added to CsgA from *E. coli* at 1:1 ratio and the mixture was incubated at 37 °C.

### Ion mobility mass spectrometry

Mass spectra and ion mobility measurements were carried out on a traveling wave ion mobility mass spectrometer, Synapt G2 HDMS instrument (Waters Corporation), equipped with a nanoflow electrospray ionization source. The source was operated in positive mode with the nESI voltage set at 1.0 to 1.2 kV, sampling cone was set to 15 V, and bias was set to 40 V. The source temperature was set to 20 °C. The traveling-wave ion mobility separator was operated at a pressure of approximately 3.3 mbar with wave height and wave velocity set at 30 V and 500 m/s, respectively. The m/z window was set from 100 to 8000 m/z with a TOF pressure of 1.5e-6 mbar. Mass spectra were analyzed using MassLynx 4.1 (https://www.waters.com/waters/en_US/MassLynx-Mass-Spectrometry-Software-/nav.htm?cid=513164&locale=en_US) and Driftscope 2.0 (https://www.waters.com/waters/library.htm?locale=en-US&cid=10010960&lid=10103987) software. CCS (Ω) measurements were externally calibrated using a database of known values in helium. We reported the SDs from replicate measurements of CCS and an additional ±3% to incorporate the errors involved with the calibration process.

### Kd (binding affinity/dissociation constant) calculation by nESI-MS

The binding affinity, often used as the dissociation constant, was calculated from nESI-MS using the relative intensity of each species from the mass spectra as described previously (59). Most nESI-MS Kd measurements involve protein and small ligand binding, where the protein–ligand complex is assumed to have the same ionization efficiency as the protein alone, due to the relatively small size of the ligand. Due to the protein–protein nature of the binding event we observed, the key assumptions implicated in most nESI-MS direct Kd measurements might not hold true. We thus carried out a calibration experiment where multiple concentrations of EC CsgA and α-synuclein were ionized (Fig. S6). Our results suggested that EC CsgA exhibited a higher ionization, transmission, and detection efficiency than those exhibited by α-synuclein in the range tested (5–40 μM). As such, we adjusted the nESI-MS intensity values while calculating Kd accordingly. In a typical protein–protein complex formation event as shown in Equation (1), the Kd is determined from the total abundance of bounded and free proteins detected at equilibrium ([P1]eq and [P2]eq), their ratio (R), and the protein initial concentrations ([P1]0 and [P2]0) as shown in Equation (2). Two different initial protein concentrations are used; triplicate data of each concentration was collected to assess error in the Kd measurements.

Formula used for the equilibrium binding of P1 (arbitrarily set as CsgA) and P2 (set as α-synuclein)is as follows:

P1P2⇌ P1+P2(Equation1)

$$Kd=\frac{{\left[P1\right]}_{eq}{\left[P2\right]}_{eq}}{{\left[P1P2\right]}_{eq}}=\frac{{\left[P2\right]}_{0}}{R}\;-\;\frac{{\left[P1\right]}_{0}}{1+R}\;(\mathrm{Equation}\;2)$$

$$\frac{{\left[P1P2\right]}_{eq}}{{\left[P1\right]}_{eq}}=\frac{Ab\left(P1P2\right)}{Ab\left(P1\right)}=R$$

## Data availability

All relevant data are in this article and its supporting information files.

## Supporting information

This article contains supporting information (31, 33, 80, 81, 82, 83)

## Conflicts of interest

The authors declare no competing financial interest.
