# Supplementary material for: Mechanistic insights into accelerated α-synuclein aggregation mediated by human microbiome-associated functional amyloids
Source: J Biol Chem. 2022 May 30;298(7):102088. doi: 10.1016/j.jbc.2022.102088 (PMC9253359; doi:10.1016/j.jbc.2022.102088)
Supplement: Supporting information [file mmc1.docx]

**Supporting Information**

**Mechanistic insights into accelerated α-synuclein aggregation mediated by human microbiome-associated functional amyloids**

Sujeet S. Bhoite^a^, Yilin Han^b^, Brandon T. Ruotolo^b*^ and Matthew R. Chapman^a*^.

^a^Department of Molecular, Cellular and Developmental Biology, University of Michigan, Ann Arbor, MI 48109-1048, USA and ^b^Department of Chemistry, University of Michigan, Ann Arbor, MI 48109-1048

*Brandon T. Ruotolo and *Matthew R. Chapman

**Email:** [bruotolo@umich.edu](mailto:bruotolo@umich.edu) and [chapmanm@umich.edu](mailto:chapmanm@umich.edu)

**This PDF file includes:**

Supporting Figures 1 to 12

Supporting Tables 1 to 4

Supporting Information (SI) References

**Fig. S1**


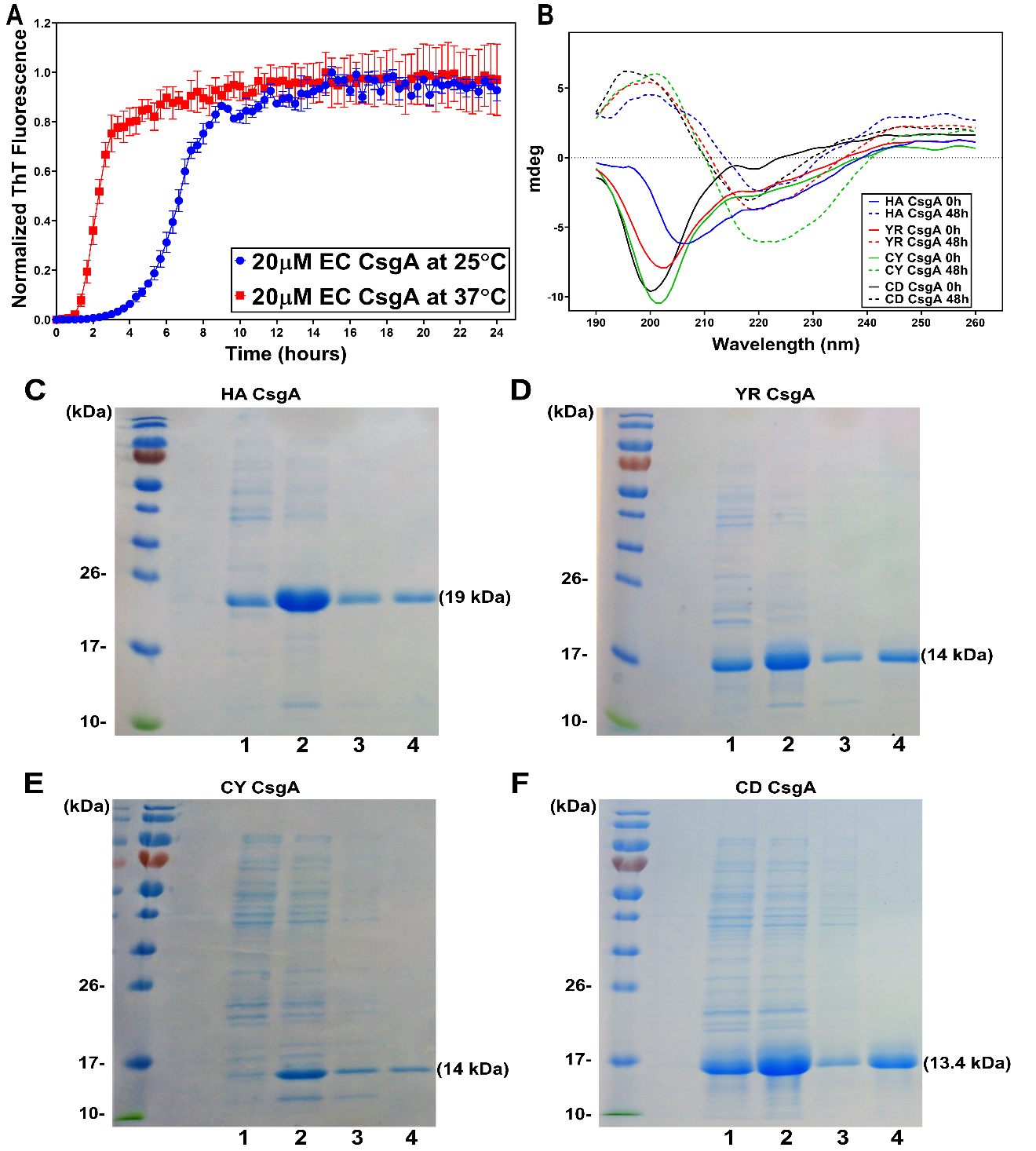


**Fig. S1. Comparison of gut bacterial CsgA homologs** (A) Aggregation kinetics of EC CsgA at 25°C and 37°C. (B) Circular dichroism spectra of gut bacterial CsgA homologs at 0h and post 48 h incubation at 37°C. (C-F) SSD-PAGE Gels of purified CsgA homologs from the gut microbiome; Lane 1- Fraction 1, Lane 2- Fraction 2, Lane 3- Fraction 3, Lane 4- Purified CsgA homologs after 30kDa cut-off column and buffer exchange treatment (see Methods for details).

**Fig. S2**


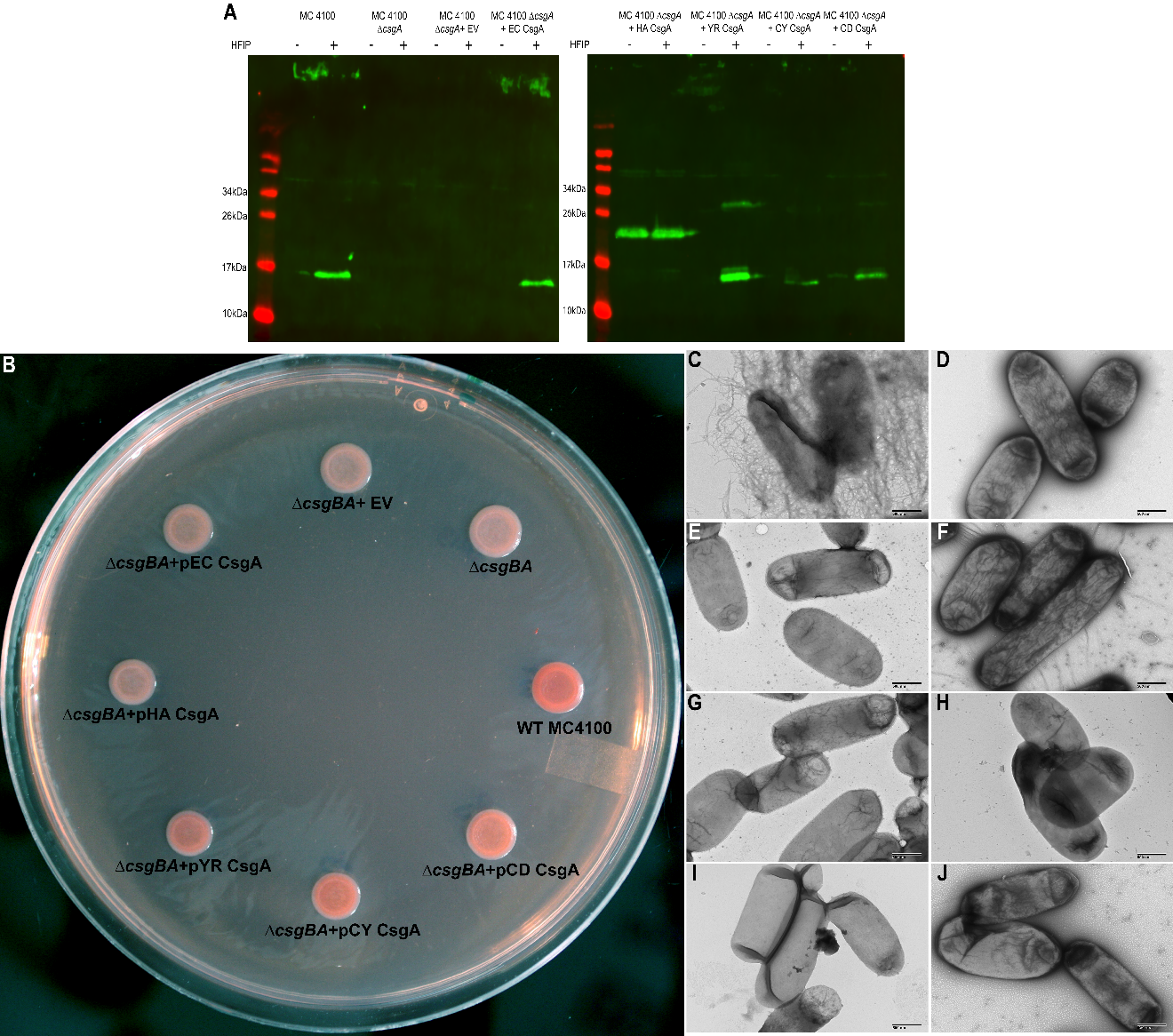


**Fig. S2. Complementation assay of gut bacterial CsgA homologs.** (A) Western blot analysis of whole cells extracts of *E. coli* MC4100 Δ*csgA* cells transformed with plasmids encoding the different CsgA homologs under the native *E. coli* *csgBAC* promoter and fused to *E. coli* CsgA sec signal and N-terminal 22 amino acids post 48 h incubation on YESCA-Congo red indicator plates and probed with polyclonal anti-CsgA antibodies. (B) *E. coli* MC4100 Δ*csgBA* cells transformed with plasmids encoding the different CsgA homologs under the native *E. coli* *csgBAC* promoter and fused to *E. coli* CsgA sec signal and N-terminal 22 amino and spotted on YESCA-Congo red indicator plates incubated for 48 h at 26°C (EV=empty vector). Representative negative-stained transmission electron micrographs of (C) WT *E. coli* MC4100, (D) *E. coli* MC4100 Δ*csgBA,* (E) *E. coli* MC4100 Δ*csgBA* + EV, (F) *E. coli* MC4100 Δ*csgBA* + pEC CsgA, (G) *E. coli* MC4100 Δ*csgBA* + pHA CsgA, (H) *E. coli* MC4100 Δ*csgBA* + pYR CsgA, (I) *E. coli* MC4100 Δ*csgBA* + pCD CsgA and (J) *E. coli* MC4100 Δ*csgBA* + pCY CsgA. (Scale bars, 500 nm).

**Fig. S3**


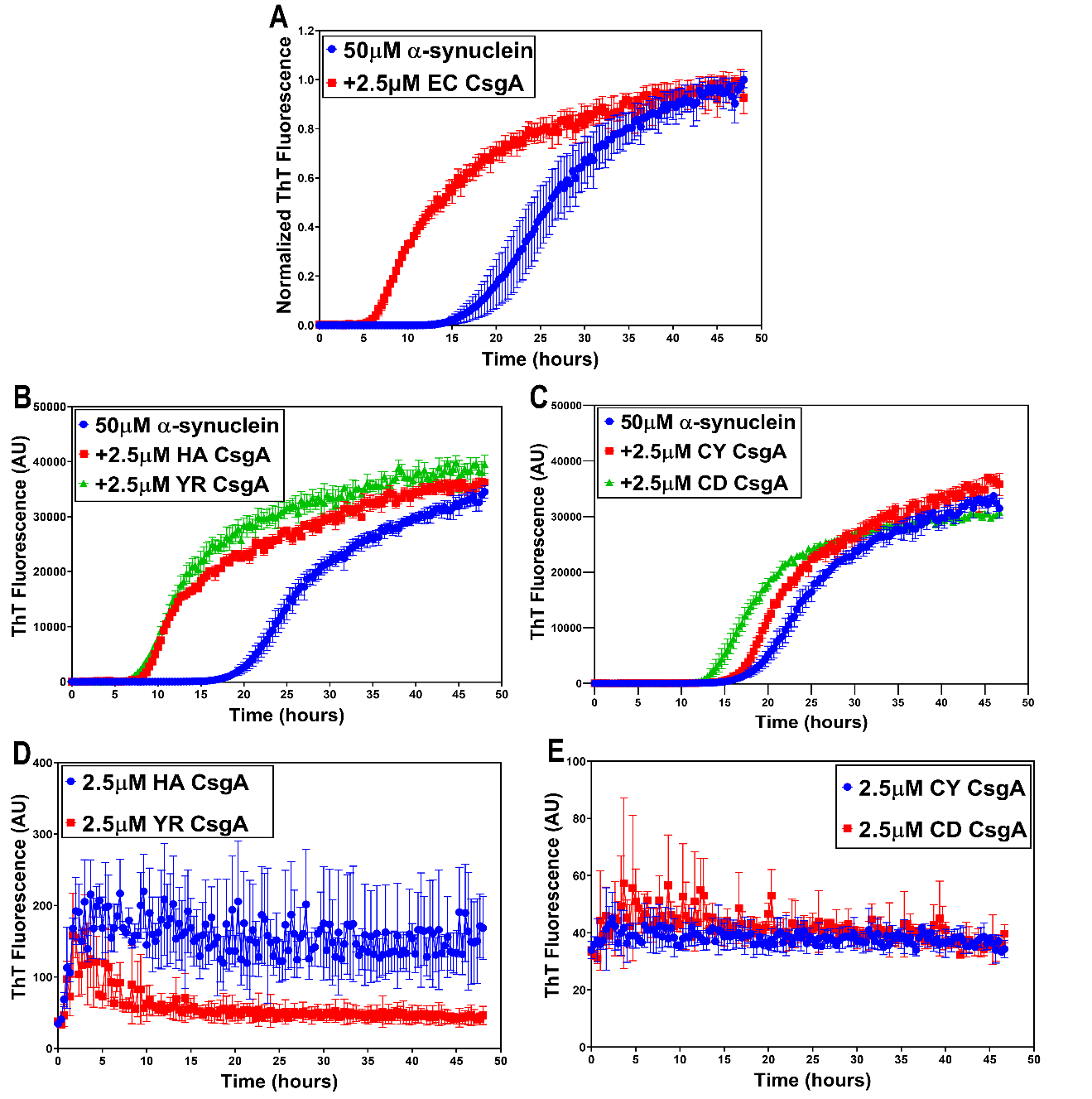


**Fig. S3. Gut bacterial CsgA homologs accelerate α-synuclein aggregation with different propensities.** Aggregation kinetics of α-synuclein alone or in presence of 2.5 µM CsgA homologs at 37⁰C. (A) α-synuclein aggregation in the presence of EC CsgA, (B-C) Raw data of α-synuclein aggregation alone or in the presence of HA CsgA, YR CsgA, CY CsgA and CD CsgA, (D-E) Raw of data 2.5 µM HA CsgA, YR CsgA CY CsgA and CD CsgA aggregation used in α-synuclein+CsgA assays. (Error bars represent SEM of three replicates).

**Fig. S4**

**
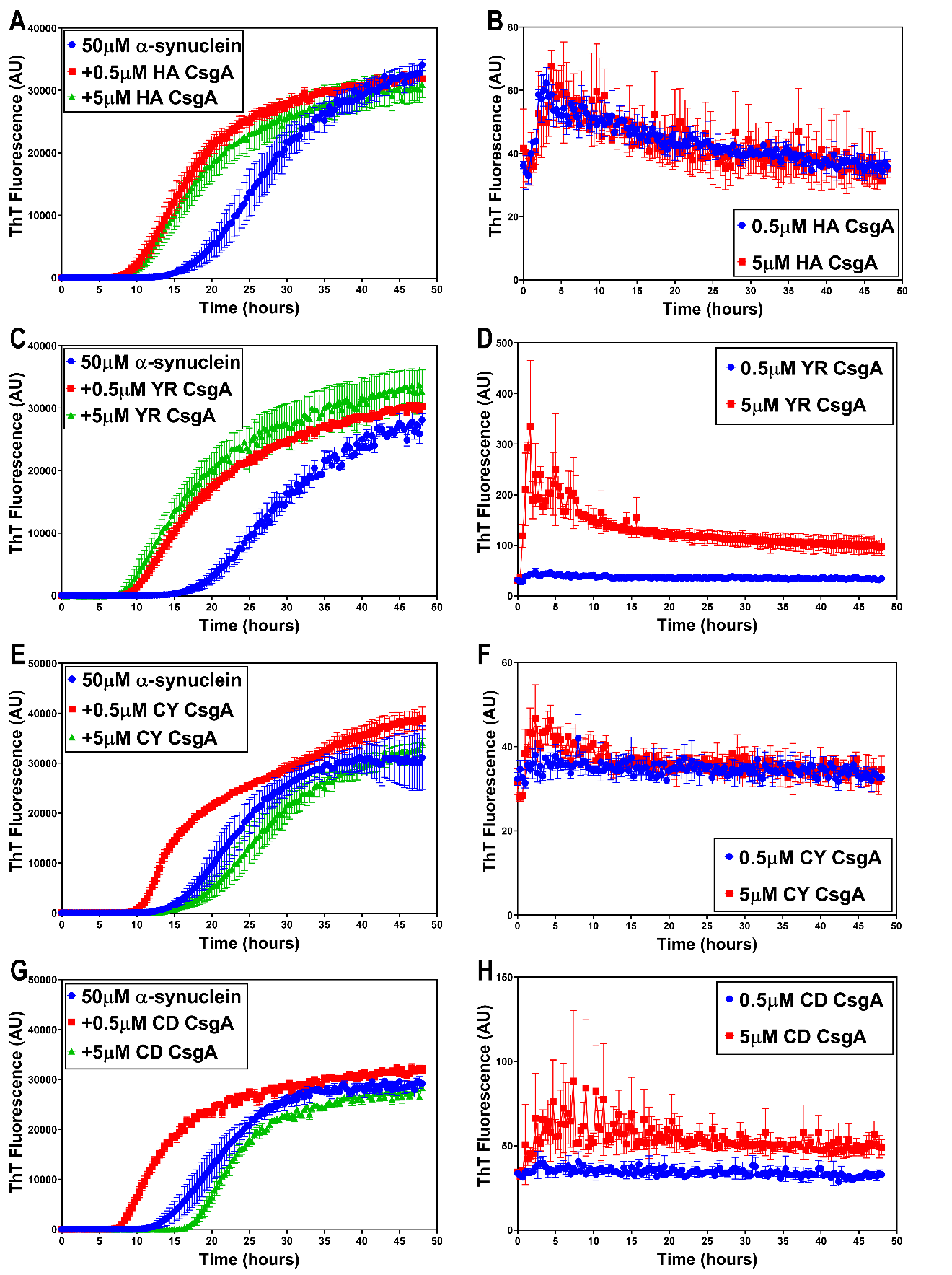
**

**Fig. S4. Aggregation kinetics of α-synuclein in presence of varying concentrations of gut bacterial CsgA homologs at 37°C.** (A) α-synuclein plus HA CsgA, (B) HA CsgA alone, (C) α-synuclein plus YR CsgA, (D) YR CsgA alone, (E) α-synuclein plus CY CsgA, (F) CY CsgA alone, (G) α-synuclein plus CD CsgA and (H) CD CsgA alone. (Error bars represent SEM of three replicates).

**Fig. S5**

**
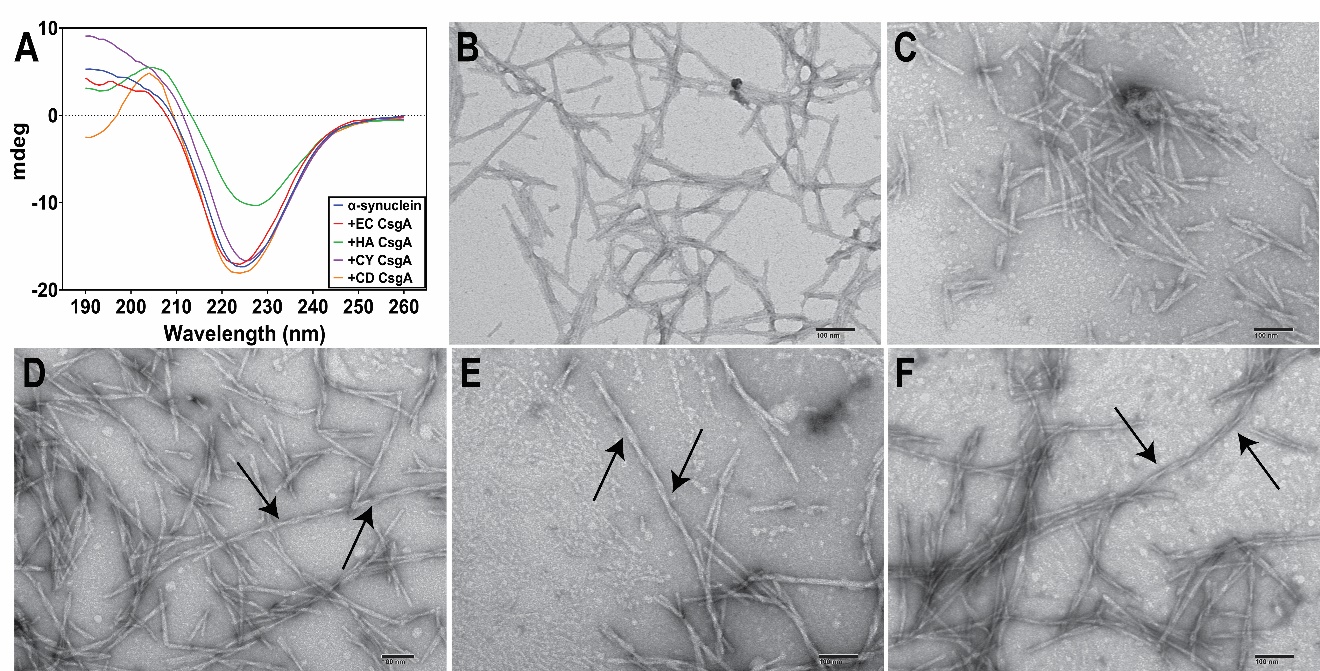
**

**Fig. S5. Fiber morphology of α-synuclein fibers generated in absence or presence of gut bacterial CsgA homologs at 37°C.** Circular dichroism spectra post 48 h incubation at 37°C of α-synuclein fibers made alone or in presence of gut bacterial CsgA homologs (A). Representative negative-stained transmission electron micrographs of α-synuclein fibers made alone or in presence of gut bacterial CsgA homologs 48 h post-aggregation (B) α-synuclein alone, (C) α-synuclein+HA CsgA, (D) α-synuclein+EC CsgA, (E) α-synuclein+CY CsgA, and (F) α-synuclein+CD CsgA. Black arrows show twisted fiber morphology observed in samples (D), (E) and (F), but not in (B) or (C). Scale bars, 100 nm.

**Fig. S6**


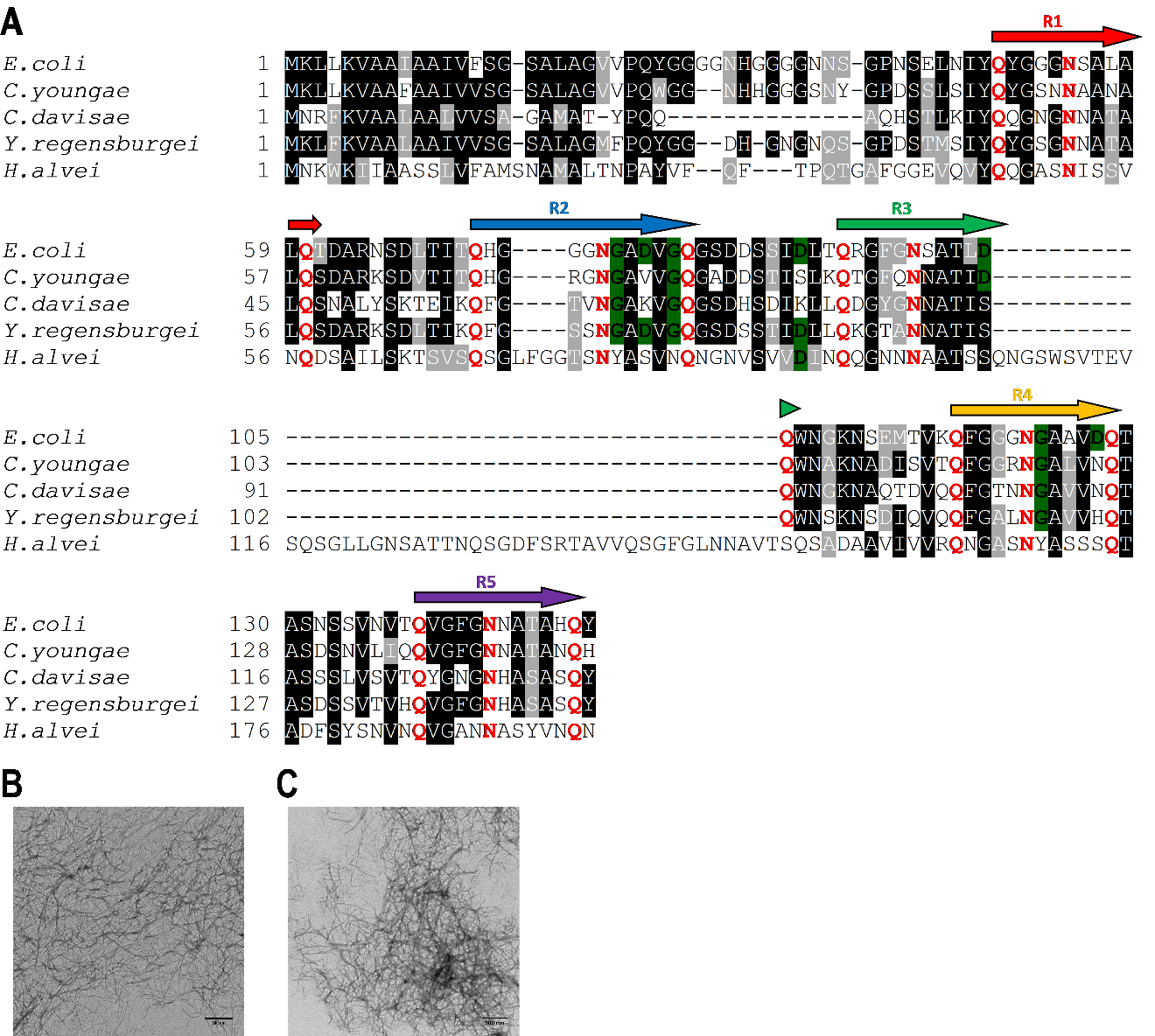


**Fig. S6. Analysis of Gatekeeper residues in CsgA homologs.** (A) Sequence alignment of CsgA homologs. The five amyloid imperfect repeat units with Q-(X)4-N-(X)5-Q motif are marked as R1 to R5. The Q and N residues in the motifs are marked in red. The gatekeeper residues are highlighted in green. In CY CsgA three gatekeeper residues are missing at positions V78, S89 and N125. In CD CsgA four gatekeeper residues are missing at positions K66, S90, K77 and N113. Negatively stained electron micrographs of CsgA fibers. Representative negative-stained transmission electron micrographs of the fibers were taken 48 h post-aggregation. (B) CY^GK^ CsgA and (C) CD^GK^ CsgA. (Scale bars, 500 nm).

**Fig. S7**


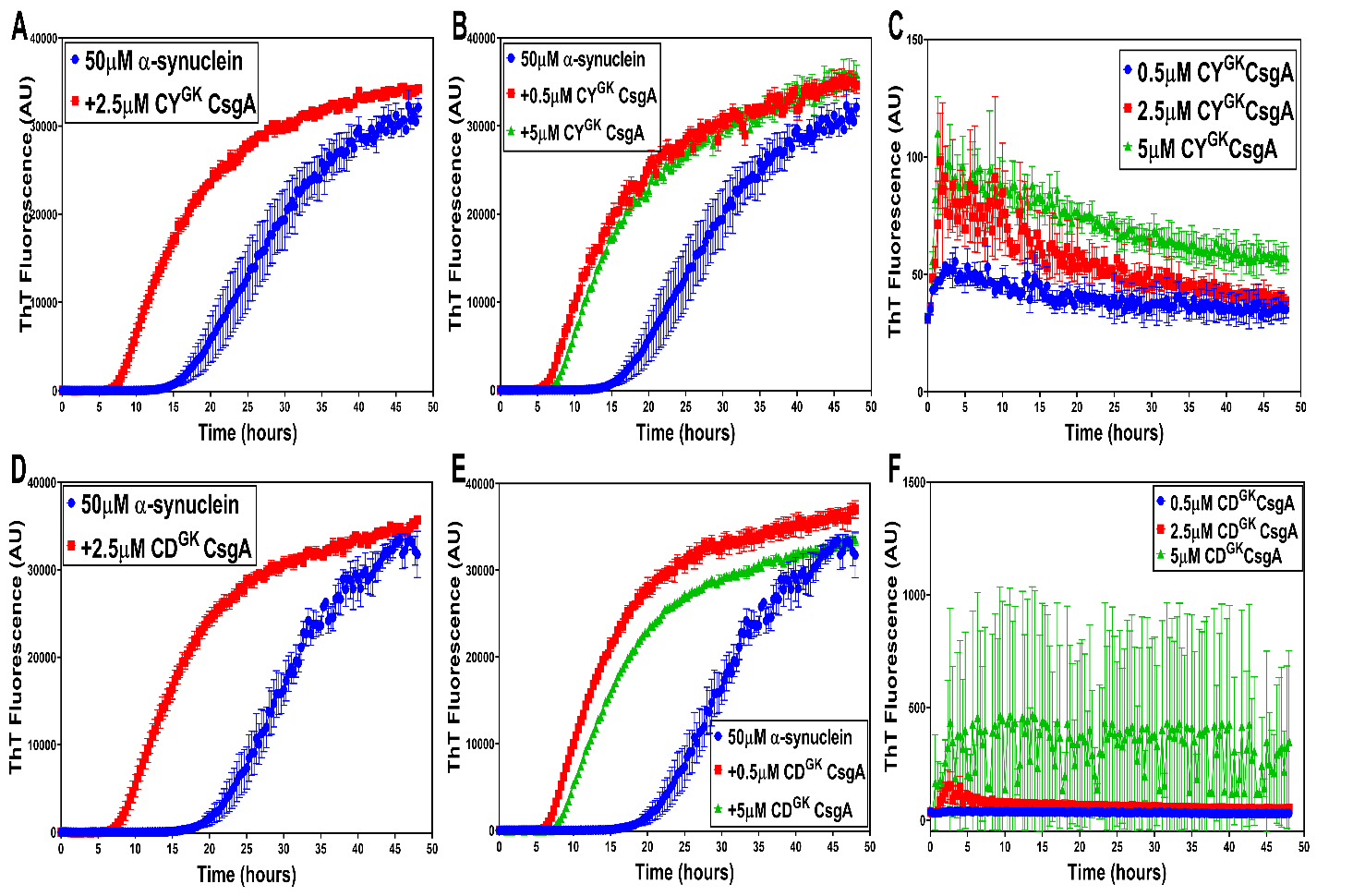


**Fig. S7. Effect of Gatekeeper residues on acceleration of α-synuclein aggregation.** Aggregation kinetics of α-synuclein alone or in presence of different concentrations of CsgA gatekeeper mutants at 37°C. (A, B) CY^GK^ CsgA plus α-synuclein, (C) CY^GK^ CsgA alone used in used in α-synuclein+CsgA assays, (D, E) CD^GK^ CsgA plus α-synuclein and (F) (C) CD^GK^ CsgA alone used in used in α-synuclein+CsgA assays (Error bars represent SEM of three replicates).

**Fig. S8**


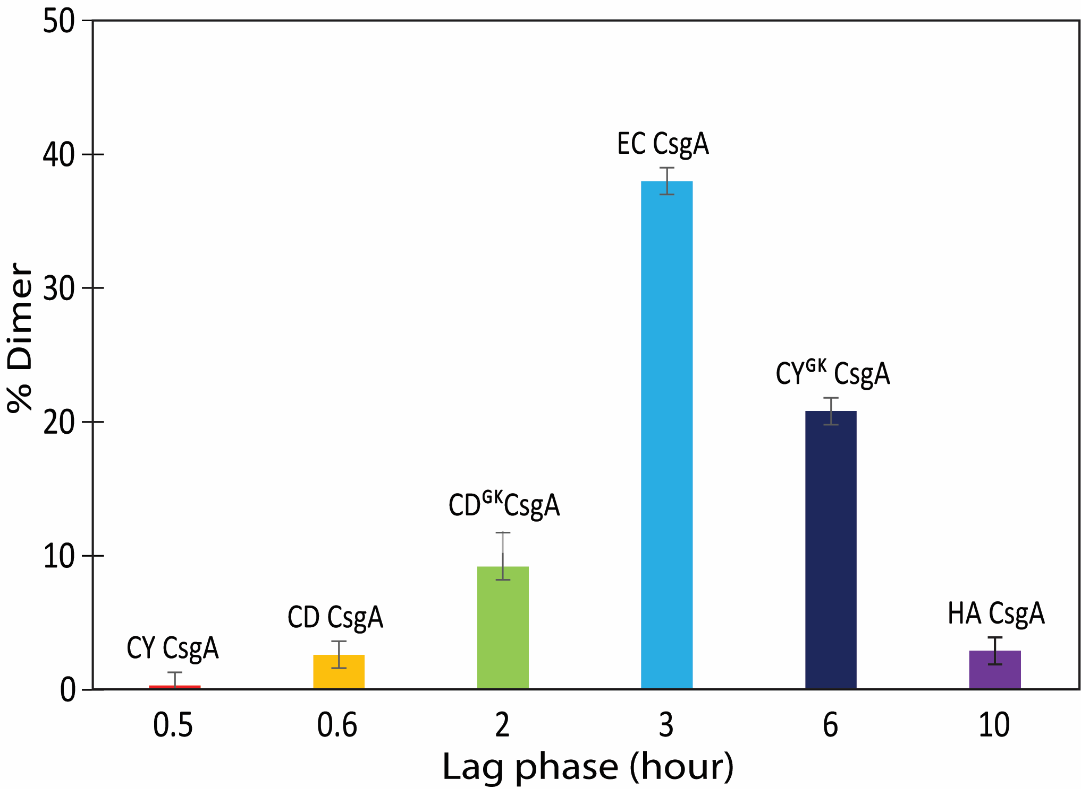


**Fig. S8. Dimer relations with aggregation kinetics**. Comparison of % dimers at 0 h for each CsgA homologs with the aggregation lag phase.

**Fig. S9**


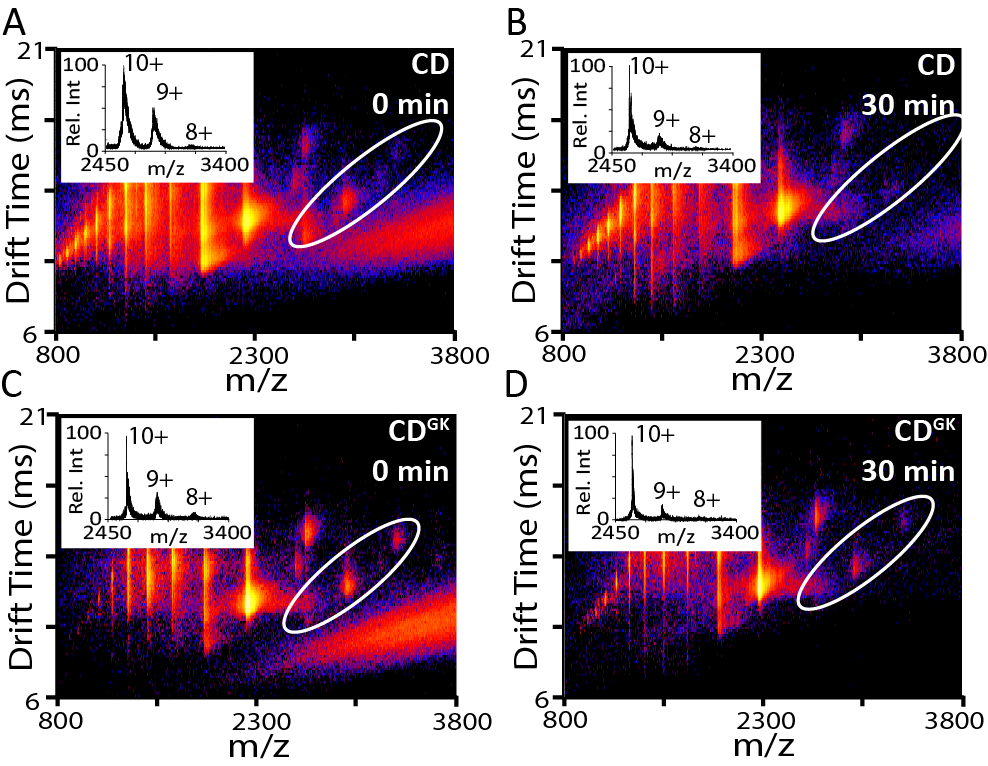


**Fig. S9. IM-MS reveals dimer relations with aggregation kinetics.** Ion mobility data for CD CsgA with enlarged mass spectra inserts featuring 8+ to 10+ charge states of dimeric CsgA (white circled) at (A) 0 minutes and (B) after 30 minutes incubation. Ion mobility data for CD^GK^ CsgA with enlarged mass spectra inserts featuring 8+ to 10+ charge states of dimeric CsgA (white circled) at (C) 0 minutes and (D) after 30 minutes incubation.

**Fig. S10**


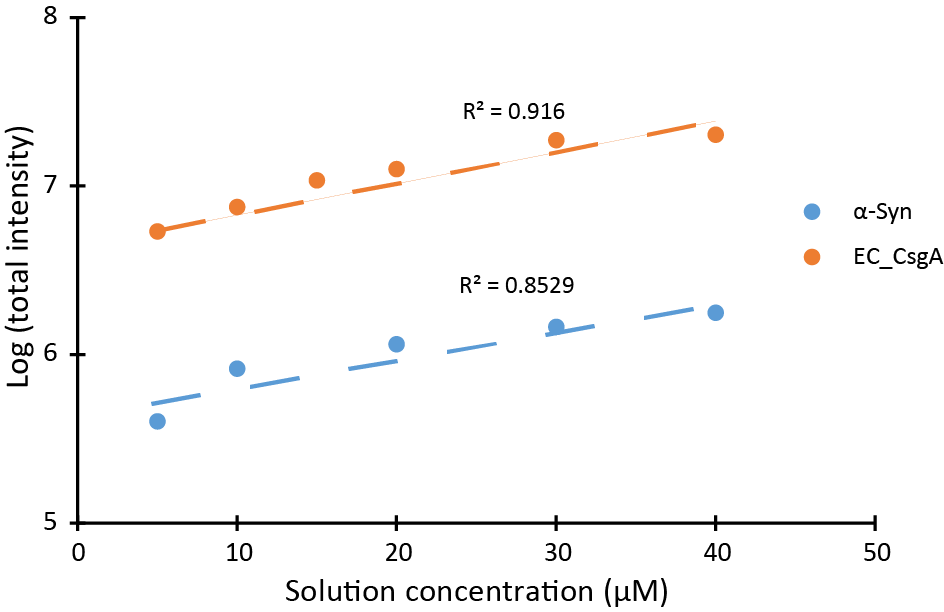


**Fig. S10. Calibration plot of logarithm of nESI-MS intensity as a function of protein solution concentration.** α-synuclein (blue) and EC CsgA (orange). These two trend-lines indicate that α-synuclein and EC CsgA do not share the same ionization efficiency as the nESI-MS intensity of the two proteins under the same concentration differs significantly. This difference was considered when calculating the Kd for α-synuclein-EC CsgA complex.

**Fig. S11**


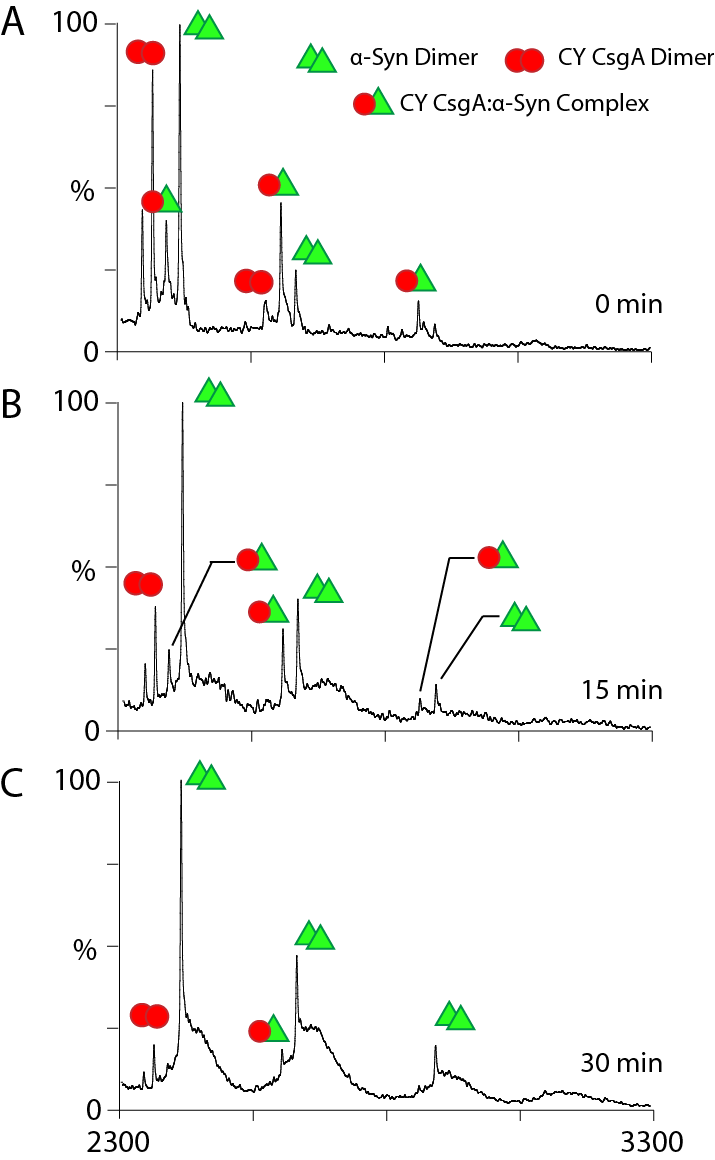


**Fig. S11. Enlarged mass spectra reveals a short-lived complex between α-synuclein and CY CsgA.** Mass spectra data for CY CsgA incubated with α-synuclein in a 1:1 molar ratio for an hour at 37°C. Dimeric CY CsgA (red double circle), dimeric α-synuclein (green double triangle) and 1:1 α-synuclein: CY CsgA complexes (green triangle and red circle) are identified and labeled. (A) at 0 min after incubation, (B) at 15 mins after incubation and (C) at 30 mins after incubation.

**Fig. S12**

**
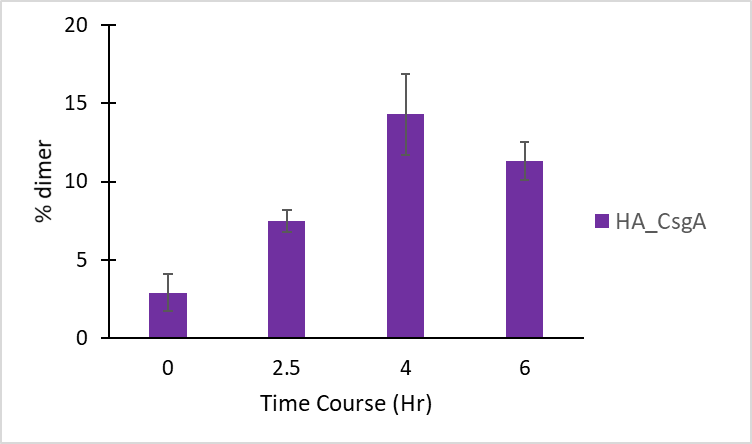
**

**Fig. S12. HA CsgA dimer % increases with time.** Comparisons of % dimer of HA CsgA obtained through IM-MS intensities values over a time course of 6 h.

**Table S1
Analysis of sequence similarity between gut bacterial CsgA homologs and EC CsgA.**The gut bacterial CsgA homologs highlighted in red were characterized in this study.


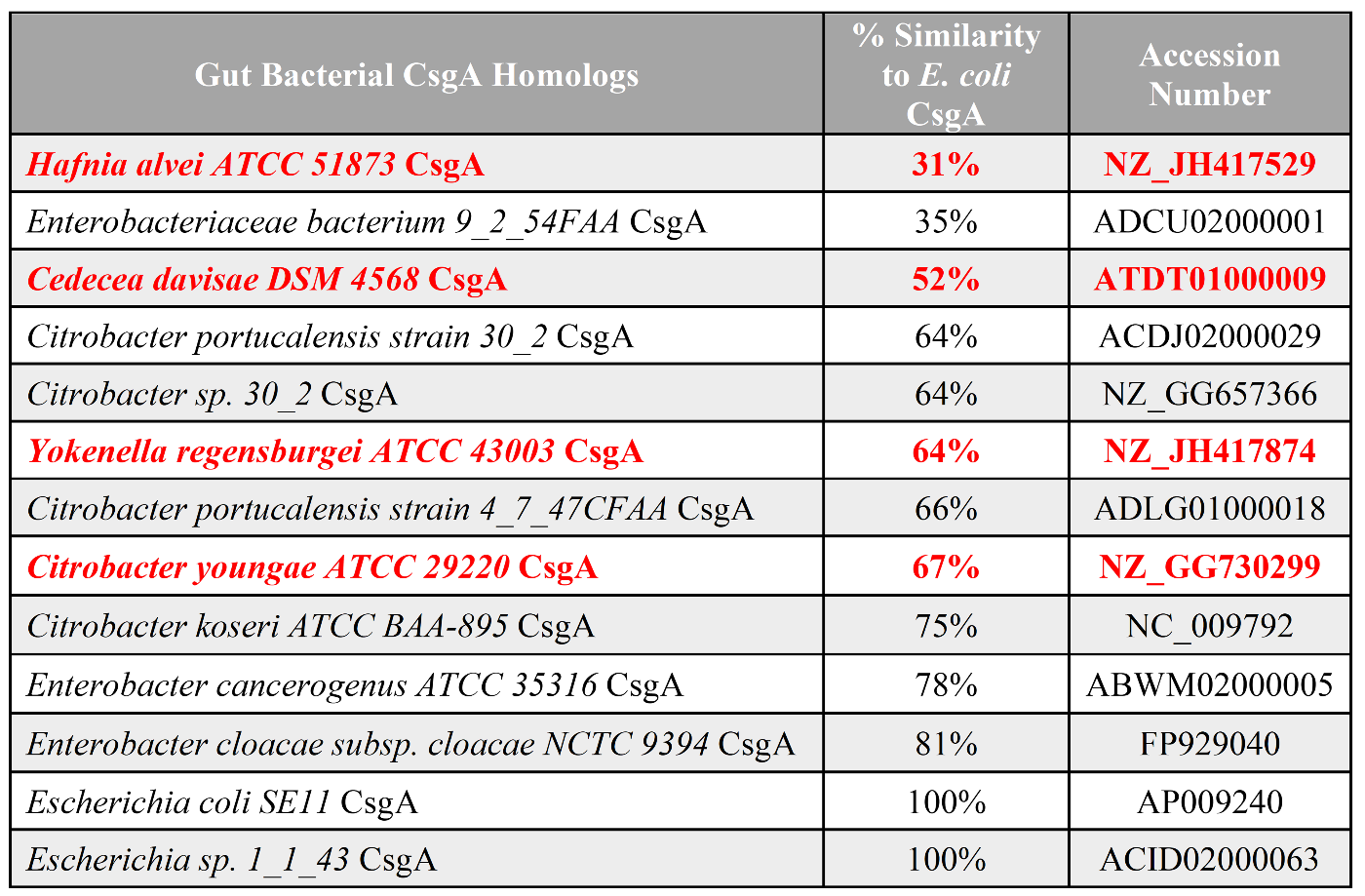


**Table S2
Percentage contribution to various secondary structural components of α-synuclein fibers** (1, 2).

| **Protein Mixture** | **% α-helix** | **% β-sheet** | **% Turn** | **% Other** |
| --- | --- | --- | --- | --- |
| α-synuclein | 0 | 39.2 | 14.8 | 46.0 |
| α-synuclein + HA CsgA | 0 | 39.8 | 14.7 | 45.5 |
| α-synuclein + EC CsgA | 0 | 39 | 14.1 | 46.9 |
| α-synuclein + CY CsgA | 0 | 39.8 | 14.9 | 45.2 |
| α-synuclein + CD CsgA | 0 | 39.7 | 14.9 | 45.4 |

**Table S3
List of oligonucleotide primers used**

| **Description** | **Sequence** | **Purpose** |
| --- | --- | --- |
| 46_CY_GB28a_Fow | 5’-CCC CAT TGC GGA ACA ACA CCC ATG GTA TAT CTC CTT CTT AAA GTT AAA CAA AAT TAT TT-3’ | To linearize pET28a with CY CsgA overhangs for Gibson assembly |
| 47_CY_GB28a_Rev | 5’-ATG CAA CAG CTA ACC AAC ACC ACC ACC ACC ACC ACC AC-3’ |  |
| 48_CYf_GB28a_Fow | 5’-TAA GAA GGA GAT ATA CCA TGG GTG TTG TTC CGC AAT GGG-3’ | To amplify CY CsgA with pET28a overhangs for Gibson assembly |
| 49_CYf_GB28a_Rev | 5’-CAG TGG TGG TGG TGG TGG TGG TGT TGG TTA GCT GTT GCA TTG TTG-3’ |  |
| 50_YRf_GB_Fow | 5’-TAA GAA GGA GAT ATA CCA TGG GTA TGT TTC CGC AAT ACG GTG-3’ | To amplify YR CsgA with pET28a overhangs for Gibson assembly |
| 51_YRf_GB_Rev | 5’-CAG TGG TGG TGG TGG TGG TGG TAT TGG CTA GCT GAC GCG T-3’ |  |
| 52_YR_GB28a_Fow | 5’-CCG TAT TGC GGA AAC ATA CCC ATG GTA TAT CTC CTT CTT AAA GTT AAA CAA AAT TAT TT-3’ | To linearize pET28a with YR CsgA overhangs for Gibson assembly |
| 53_YR_GB28a_Rev | 5’-ACG CGT CAG CTA GCC AAT ACC ACC ACC ACC ACC ACC ACT-3’ |  |
| 54_CD_GB28a_Fow | 5’-GCC TGC TGC GGG TAA GTA GCC ATG GTA TAT CTC CTT CTT AAA GTT AAA CAA AAT TAT TT-3’ | To linearize pET28a with CD CsgA overhangs for Gibson assembly |
| 55_CD_GB28a_Rev | 5’-ACG CTT CAG CTT CCC AGT ACC ACC ACC ACC ACC ACC ACT-3’ |  |
| 56_CDf_GB28a_Fow | 5’-TAA GAA GGA GAT ATA CCA TGG CTA CTT ACC CGC AGC-3’ | To amplify CD CsgA with pET28a overhangs for Gibson assembly |
| 57_CDf_GB28a_Rev | 5’-CAG TGG TGG TGG TGG TGG TGG TAC TGG GAA GCT GAA GCG T-3’ |  |
| 58_HA_GB28a_Fow | 5’-GCT GGG TTA GTT AAC ATG GTA TAT CTC CTT CTT AAA GTT AAA CAA AAT TAT TTC TAG AGG-3’ | To linearize pET28a with HA CsgA overhangs for Gibson assembly |
| 59_HA_GB28a_Rev | 5’-CCT CAT ACG TTA ATC AGA ACC ACC ACC ACC ACC ACC ACT-3’ |  |
| 60_HAf_GB28a_Fow | 5’-TAA GAA GGA GAT ATA CCA TGT TAA CTA ACC CAG CTT ACG TTT TCC AAT TCA CAC C-3’ | To amplify HA CsgA with pET28a overhangs for Gibson assembly |
| 61_HAf_GB28a_Rev | 5’-CAG TGG TGG TGG TGG TGG TGG TTC TGA TTA ACG TAT GAG GCG TTG T-3’ |  |
| 66_T176A_A177T_CY_CsgA | 5’-GGT AAC GGG GCT GAT GTC GGC CAG GGC G-3’ | To introduce gatekeeper residues in CY CsgA |
| 67_A208G_G209A_CY_CsgA | 5’-CTG ATG ACA GCA CCA TTG ATC TGA AAC AGA CTG GCT TCC AGA ACA ATG-3’ |  |
| 68_A316G_CY_CsgA | 5’-CAA CGG TGC GCT GGT TGA TCA GAC TGC TTC TG-3’ |  |
| 69_A142G_A144T_CD_CsgA | 5’-CCG TTA ACG GCG CCG ATG TGG GCC AGG GTT C-3’ | To introduce gatekeeper residues in CD CsgA |
| 70_A214G_G215A_C216T_  CD_CsgA | 5’-CTA TGG CAA CAA TGC AAC CAT CGA TCA GTG GAA TGG TAA AAA TGC GC-3’ |  |
| 71_A283G_C285T_CD_CsgA | 5’-CAA CGG AGC CGT CGT TGA TCA GAC TGC TTC GAG C-3’ |  |
| 72_A175G_A177T_CD_CsgA | 5’-GGT TCT GAC CAC AGC GAT ATT GAT TTG CTG CAA GAC GGC T-3’ |  |
| 73_pLR5_HA_Fow | 5’-TTA ATC AGA ACT AAG GAT CCT CTA GAG TCG AC-3’ | To linearize pLR5 with HA CsgA overhangs for Gibson assembly |
| 74_pLR5_HA_Rev | 5’-ACC TCA CCA TTT GGG CCG CTA TTA TTA CCG-3’ |  |
| 75_HAf_pLR5_Fow | 5’-GCC CAA ATG GTG AGG TGC AGG TCT AT-3’ | To amplify HA CsgA with pLR5 overhangs for Gibson assembly |
| 76_HAf_pLR5_Rev | 5’-AGA GGA TCC TTA GTT CTG ATT AAC GTA TGA GGC GTT G-3’ |  |
| 77_pLR5_CY_Fow | 5’-TAA CCA ACA CTA AGG ATC CTC TAG AGT CGA C-3’ | To linearize pLR5 with CY CsgA overhangs for Gibson assembly |
| 78_pLR5_CY_Rev | 5’-AGC GAA GAA TTT GGG CCG CTA TTA TTA CC-3’ |  |
| 79_CYf_pLR5_Fow | 5’-GCC CAA ATT CTT CGC TGA GTA TCT ACC AAT ATG G-3’ | To amplify CY CsgA with pLR5 overhangs for Gibson assembly |
| 80_CYf_pLR5_Rev | 5’-AGG ATC CTT AGT GTT GGT TAG CTG TTG CAT TG-3’ |  |
| 81_pLR5_CD_Fow | 5’-TCC CAG TAC TAA GGA TCC TCT AGA GTC G-3’ | To linearize pLR5 with CD CsgA overhangs for Gibson assembly |
| 82_pLR5_CD_Rev | 5’-AGG GTT GAA TTT GGG CCG CTA TTA TTA CCG-3’ |  |
| 83_CDf_pLR5_Fow | 5’-GCC CAA ATT CAA CCC TGA AAA TTT ATC AGC AGG G-3’ | To amplify CD CsgA with pLR5 overhangs for Gibson assembly |
| 84_CDf_pLR5_Rev | 5’-AGG ATC CTT AGT ACT GGG AAG CTG AAG CG-3’ |  |
| 85_pLR5_YR_Fow | 5’-AGC CAA TAC TAA GGA TCC TCT AGA GTC GAC CTG-3’ | To linearize pLR5 with YR CsgA overhangs for Gibson assembly |
| 86_pLR5_YR_Rev | 5’-CAT CGT AGA ATT TGG GCC GCT ATT ATT ACC-3’ |  |
| 87_YRf_pLR5_Fow | 5’-GGC CCA AAT TCT ACG ATG TCC ATT TAC CAG TAC G-3’ | To amplify YR CsgA with pLR5 overhangs for Gibson assembly |
| 88_YRf_pLR5_Rev | 5’-AGG ATC CTT AGT ATT GGC TAG CTG ACG CG-3’ |  |

**Table S4
Strains and plasmids used in this study**

| **Strains** | **Relevant characteristics** | **References** |
| --- | --- | --- |
| LSR10 | MC4100 *ΔcsgA* | (3) |
| LSR13 | MC4100 *ΔcsgBA* | (4) |
| NEB 3016 | T7 Express *I^q^* Competent *E. coli* | NEB Inc. |
|  | | |
| **Plasmids** | **Relevant characteristics** | **References** |
| pLR2 | Control vector containing *E. coli* *csgBA* promoter | (3) |
| pLR5 | *E. coli csgA* sequence cloned in pLR2 | (5) |
| pLR2_HA | HA CsgA sequence cloned in pLR2 | This study |
| pLR2_YR | YR CsgA sequence cloned in pLR2 | This study |
| pLR2_CD | CD CsgA sequence cloned in pLR2 | This study |
| pLR2_CY | CY CsgA sequence cloned in pLR2 | This study |
| pET28a | IPTG inducible expression vector | NEB Inc. |
| pET11d | IPTG inducible expression vector | NEB Inc. |
| pNH11 | C-terminal His6 tagged *E. coli csgA* cloned into NcoI/BamHI site in pET11d | (6) |
| pET28a_HA | C-terminal His6 tagged HA CsgA cloned in pET28a | This study |
| pET28a_YR | C-terminal His6 tagged YR CsgA cloned in pET28a | This study |
| pET28a_CD | C-terminal His6 tagged CD CsgA cloned in pET28a | This study |
| pET28a_CY | C-terminal His6 tagged CY CsgA cloned in pET28a | This study |
| pET28a_CY^GK^ | C-terminal His6 tagged CY CsgA with gatekeeper mutations cloned in pET28a | This study |
| pET28a_CD^GK^ | C-terminal His6 tagged CD CsgA with gatekeeper mutations cloned in pET28a | This study |

**SI References**

1. Micsonai, A., Wien, F., Bulyáki, É., Kun, J., Moussong, É., Lee, Y.-H., Goto, Y., Réfrégiers, M., and Kardos, J. (2018) BeStSel: a web server for accurate protein secondary structure prediction and fold recognition from the circular dichroism spectra. *Nucleic Acids Research*. **46**, W315–W322

2. Micsonai András, Wien Frank, Kernya Linda, Lee Young-Ho, Goto Yuji, Réfrégiers Matthieu, and Kardos József (2015) Accurate secondary structure prediction and fold recognition for circular dichroism spectroscopy. *Proceedings of the National Academy of Sciences*. **112**, E3095–E3103

3. Chapman, M. R., Robinson, L. S., Pinkner, J. S., Roth, R., Heuser, J., Hammar, M., Normark, S., and Hultgren, S. J. (2002) Role of <em>Escherichia coli</em> Curli Operons in Directing Amyloid Fiber Formation. *Science*. **295**, 851

4. Barnhart, M. M., and Chapman, M. R. (2006) Curli Biogenesis and Function. *Annu. Rev. Microbiol.* **60**, 131–147

5. Hammer, N. D., Schmidt, J. C., and Chapman, M. R. (2007) The curli nucleator protein, CsgB, contains an amyloidogenic domain that directs CsgA polymerization. *Proc Natl Acad Sci USA*. **104**, 12494

6. Hammer, N. D., McGuffie, B. A., Zhou, Y., Badtke, M. P., Reinke, A. A., Brännström, K., Gestwicki, J. E., Olofsson, A., Almqvist, F., and Chapman, M. R. (2012) The C-Terminal Repeating Units of CsgB Direct Bacterial Functional Amyloid Nucleation. *Journal of Molecular Biology*. **422**, 376–389
